# Lumped-Parameter Circuit Platform for Simulating Typical Cases of Pulmonary Hypertensions from Point of Hemodynamics

**DOI:** 10.1007/s12265-020-09953-y

**Published:** 2020-01-13

**Authors:** Hong Tang, Ziyin Dai, Miao Wang, Binbin Guo, Shunyu Wang, Jiabin Wen, Ting Li

**Affiliations:** 1 School of Biomedical EngineeringDalian University of Technology, Dalian City, China; 2 The Second Hospital of Dalian Medical University, Dalian City, China; 3 School of Information and Communication EngineeringDalian Minzu University, Dalian City, China

**Keywords:** Pulmonary hypertension, Human circulation system, Distal pulmonary artery stenosis, Left ventricular diastolic dysfunction, Ventricular septal defect, Mitral stenosis, Hemodynamic modeling

## Abstract

Pulmonary hypertension (PH) presents unusual hemodynamic states characterized by abnormal high blood pressure in pulmonary artery. The objective of this study is to simulate how the hemodynamics develops in typical PH cases without treatment. A lumped-parameter circuit platform of human circulation system is set up to simulate hemodynamic abnormalities of PH in different etiologies and pathogenesis. Four typical cases are considered, which are distal pulmonary artery stenosis, left ventricular diastolic dysfunction, ventricular septal defect, and mitral stenosis. The authors propose regulation laws for chambers and vessels to adapt the abnormal hemodynamic conditions for each PH case. The occurrence and development of each PH case are simulated over time using the lumped-parameter circuit platform. The blood pressure, blood flow, pressure-volume relations for chambers and vessels are numerically calculated for each case of PH progression. The model results could be a quite helpful to understand the hemodynamic mechanism of typical PHs.

**Electronic supplementary material:**

The online version of this article (10.1007/s12265-020-09953-y) contains supplementary material, which is available to authorized users.

**Figure:**
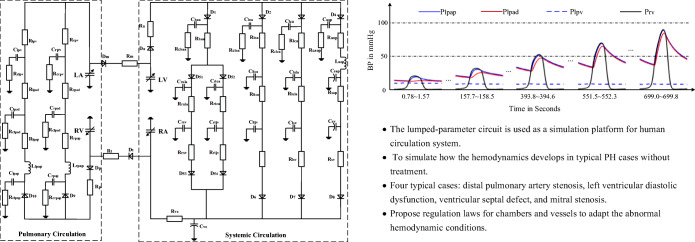
Graphical Abstract

## Background

Pulmonary hypertension (PH) is a general term to describe groups of clinical syndromes characterized by high pressure in the lungs caused by different etiologies and pathogenesis. At sea level, a cardiac output of 5 to 6 L/min is associated with a pulmonary artery pressure of about 20/12 mmHg. PH is considered if a mean pulmonary artery pressure is greater than 25 mmHg. The World Health Organization (WHO) defines five groups of PH based on different causes. They are referred to as PH WHO groups [1]. The first group is pulmonary arterial hypertension (PAH), caused by narrowing, thickening, and stiffening of pulmonary arteries. The second group is PH due to left heart diseases. In this group, there are problems in the manner how the heart squeezes or relaxes, or problems with the valves on the left side of the heart. The third group is PH due to lung diseases. The fourth group is PH due to chronic blood clots in the lungs. The fifth group is PH due to unknown causes. PH lacks distinctive clinical manifestations in the early stage. No matter which group one patient is in, PH is a serious disease. If PH is not treated timely, pulmonary artery pressures would reach systemic levels, right heart failure becomes inevitable [2]. Since any group of PH can be reflected by the abnormal hemodynamics in the right heart and lungs, it is necessary to understand how the hemodynamic changes over time therein. At present, right heart catheterization that directly measures blood pressure in the right heart and lungs is the ‘gold standard’ operation for diagnosis and assessment of PH [3].

Due to the numerous interactions within the cardiovascular system, it is often unclear how a change in a cardiac or vascular parameter affects the patient’s overall hemodynamics. Mathematical models and computer simulations may become cheap and convenient ways to understand the causes and development of abnormal hemodynamics in systemic and pulmonary circulation system. Various models have been proposed for circulation hemodynamic simulation. A circuit model was built for heart failure, which found a decrease in left ventricular blood pressure and cardiac output, and a significant change in the pressure-volume (P-V) loop of left ventricle (LV) [4–6]. Korurek et al. modeled severe aortic valve stenosis by increasing the value of the resistance to the aortic valve in the analog circuit model [7], in which a remarkable increase in LV systolic blood pressure and aortic pressure mean gradient, and decrease in aortic systolic blood pressure was consequently observed. In addition, mitral stenosis [8], mitral regurgitation, and aortic regurgitation [9], causing the abnormal hemodynamics in the cardiovascular system, were also studied by the computer model. In [10], two causes that lead to left ventricular diastolic dysfunction were discussed. Impaired left ventricular active relaxation (IR-type) was modeled by changing the activation function of LV. Increased passive stiffness (R-type) was modeled by increasing diastolic stiffness of LV wall and septum. The simulation results showed that abnormal LV diastolic performance alone can result in decreased LV and right ventricular (RV) systolic performance [10]. Besides, Korurek et al. simulated Eisenmenger syndrome with ventricular septal defect [11]. It was found that there was a remarkable increase in the pulmonary artery pressure and RV pressure, but decrease in LV pressure, aortic pressure, aortic flow and pulmonary compliance.

PH is a final common hemodynamic consequence of multiple etiologies and diverse mechanisms. In this study, the authors deal with chronic PH and set up a lumped-parameter circuit network as a platform for simulating four typical cases of PH, including PH caused by distal pulmonary artery stenosis (DPAS), left ventricular diastolic dysfunction (LVDD), ventricular septal defect (VSD), and mitral stenosis (MS). The simulations show successful occurrence and development of these PH cases without treatment.

## A Lumped-Parameter Platform for Normal Human Circulation System

**Fig. 1:**
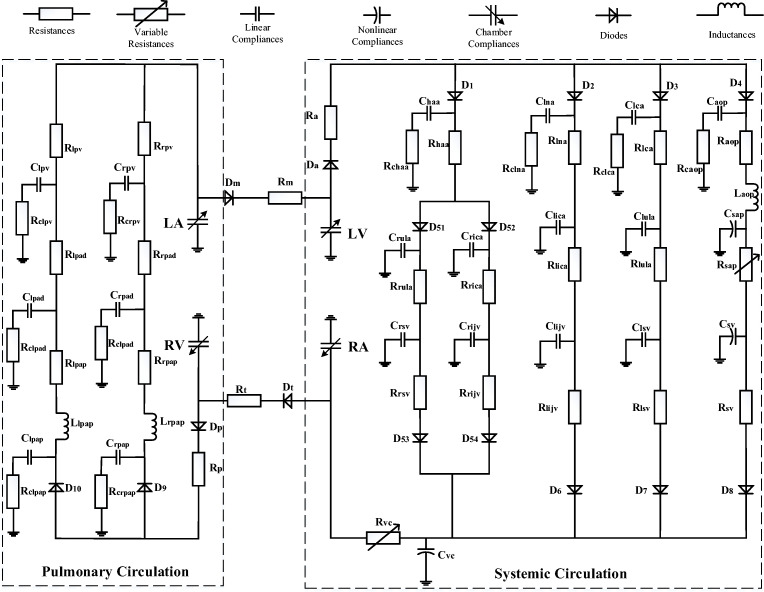
Lumped-parameter circuit platform for normal human circulation system. R: resistances; C: compliances; L: inductances; D: valves; LV: left ventricle; LA: left atrium; RV: right ventricle; RA: right atrium. Full name for the abbreviations used in subscripts: Dm-mitral valve; Da-aortic valve; Dt-tricuspid valve; Dp-pulmonary valve; haa-head and arm artery; lna-left neck artery; lca-left clavicular artery; aop-proximal aorta; rula-right upper limb artery; rica-right internal carotid artery; lica-left internal carotid artery; lula-left upper limb artery; sap-proximal systemic artery; rsv-right subclavian vein; rijv-right internal jugular vein; lijv-left internal jugular vein; lsv-left subclavian vein; sv-systemic veins; vc-vena cava; lpap-left proximal pulmonary artery; rpap-right proximal pulmonary artery; lpad-left distal pulmonary artery; rpad-right distal pulmonary artery; lpv-left pulmonary veins; rpv-right pulmonary veins; c-viscoelastic resistance. D_1_, D_2_, D_3_, D_4_, D_51_, D_52_, D_53_, D_54_, D_6_, D_7_, D_8_, D_9_, and D_10_ are diodes for valves

Previous studies have clearly disclosed that there is general equivalence between the blood flow in circulation system and the current flow in analog circuit [12–15]. The blood pressure and blood flow are equivalent to the voltage and charge flow. The resistance of blood flow is equivalent to the electronic resistance. The inertia of blood flow can be modeled by the inductance. Inflow and outflow blood to vessel are similar to charging and discharging to linear or nonlinear capacitance. Blood pumping of a heart chamber can be simulated by a nonlinear voltage source with respect to volume and time. Valves in heart and vessels are like diodes. Therefore, an improved circuit model for human circulation system is proposed in this study and taken as a platform to simulate four typical cases of PH, see Fig. 1. The P-V relation of a segment of vein or artery is generally modeled by a three-element Windkessel: resistance, compliance, and inductance. The initial values of the elements in the model are given in Appendix A.

### Ventricular Model

The ventricular model in this paper is based on the work of Chung et al. [13]. Each ventricle is characterized as a time-varying elastance function that is controlled by end-systolic P-V relation (ESPVR), end-diastolic P-V relation (EDPVR), and a time-varying activation function. From a physiological point of view, great sympathetic tone increases myocardial elastance and shortens ventricular systole. Therefore, a rising in the sympathetic efferent discharge frequency, *F*_*con*_, increases the maximum elastance [14]. For example, the blood pressure in the left ventricle,*P*_*lv*_, is a function of volume *V*_*lv*_, time *t* and *F*_*con*_,

1 $$ {P}_{lv}\left({V}_{lv},t,{F}_{con}\right)={e}_{lv}\left(t,{F}_{con}\right)\times {P}_{lv\_ ES}\left({V}_{lv},{F}_{con}\right)+\left(1-{e}_{lv}\left(t,{F}_{con}\right)\right)\times {P}_{lv\_ ED}\left({V}_{lv}\right), $$

2a $$ {P}_{lv\_ ES}\left({V}_{lv},{F}_{con}\right)=a\left({F}_{con}\right)\times {E}_{es\_ lv}\times \left({V}_{lv}-{V}_{d\_ lv}\right), $$

2b $$ {P}_{lv\_ ED}\left({V}_{lv}\right)={M}_{0\_ lv}\times \mid \exp \left[{\lambda}_{lv}\left({V}_{lv}-{V}_{0\_ lv}\right)\right]-1\mid, $$

where *P*_*lv* _ *ES*_(*V*_*lv*_, *F*_*con*_) represents the ESPVR and *P*_*lv* _ *ED*_(*V*_*lv*_) represents the EDPVR. *V*_*d* _ *lv*_ is the constant volume. *E*_*es* _ *lv*_ is the end-systolic elastance. *V*_0 _ *lv*_ is the volume intercept of EDPVR, *M*_0 _ *lv*_ is the pressure intercept, and *λ*_*lv*_ is the empirical constant. In addition, a rising in *F*_*con*_ also shortens the ventricular systolic period, so Lu and Clark et al. [14] modified the ventricular activation function that is a function of *F*_*con*_, and the activation function *e*_*lv*_(*t*, *F*_*con*_) consists of four Gaussian functions,

3 $$ {e}_{lv}\left(t,{F}_{con}\right)=\sum \limits_{i=1}^4{A}_i\times \exp\;\left[-{\left(\frac{b\left({F}_{con}\right)\ast {\left[t\right]}_T-{C}_i}{B_i}\right)}^2\right], $$

where [*t*]_*T*_ is the operation to get the remainder after division of *t* by *T*. *T* is the cardiac cycle duration. So, [*t*]_*T*_ must be equal to or greater than 0 and less than *T*. *a*(*F*_*con*_) and *b*(*F*_*con*_) are functions of *F*_*con*_,

4a $$ a\left({F}_{con}\right)={a}_{min}+{K}_a\times {F}_{con}, $$

4b $$ b\left({F}_{con}\right)={b}_{min}+{K}_b\times {F}_{con}, $$

*a*_*min*_ and *b*_*min*_ are the minimum values of the functions *a* and *b*. *K*_*a*_ and *K*_*b*_ are scaling factors. Those parameters are shown in Table 1.

**Table 1:** Parameters for control of ventricle contractility [15]

Ventricle contractility			
*a*_min_	*b*_min_	*K*_*a*_	*K*_*b*_
− 2	0.7	7	0.5

### Atrial Model

Based on the works [13, 14], the atrial model is characterized as a time-varying elastic function that is controlled by ESPVR, EDPVR, and a time-varying activation function. For example, the blood pressure in the left atrium,*P*_*la*_, is a function of volume *V*_*la*_ and time *t* [14, 15],

5a $$ {P}_{la}\left({V}_{la},t\right)={e}_{la}(t)\times {P}_{la\_ ES}\left({V}_{la}\right)+\left(1-{e}_{la}(t)\right)\times {P}_{la\_ ED}\left({V}_{la}\right), $$

5b $$ {P}_{la\_ ES}\left({V}_{la}\right)={E}_{es\_ la}\times \left({V}_{la}-{V}_{d\_ la}\right), $$

5c $$ {P}_{la\_ ED}\left({V}_{la}\right)={M}_{\_\_ la}\times \mid \exp\;\left[{\lambda}_{la}\left({V}_{la}-{V}_{\_\_ la}\right)\right]-1\mid . $$

The activation function *e*_*la*_(*t*) is fitted by one Gaussian function [15],

6 $$ {e}_{la}(t)={A}_0\times \exp\;\left[-0.5\times {\left(\frac{{\left[t\right]}_T-{C}_0}{B_0}\right)}^2\right]. $$

The right ventricular model and the right atrial model are like those of left ventricle and left atrium. The parameters for the models are shown in Table 2 and Table 3. Therefore, the blood pressure of the four chambers *P*_*lv*_(*V*_*lv*_, *t*), *P*_*la*_(*V*_*la*_, *t*), *P*_*rv*_(*V*_*rv*_, *t*)_,_ and *P*_*ra*_(*V*_*ra*_, *t*) can be modeled with respect to volume and time.

**Table 2:** Parameters of the ventricular and atrial model

Parameter	Value	Parameter	Value
*E*_*es* _ *lv*_ mmHg/ml [15]	4.3	*E*_*es* _ *rv*_ mmHg/ml	0.8
*E*_*es* _ *la*_ mmHg/ml	0.3	*E*_*es* _ *ra*_ mmHg/ml	0.3
*M*_0 _ *lv*_ mmHg [15]	1.7	*M*_0 _ *rv*_ mmHg [15]	0.67
*M*_0 _ *la*_ mmHg [15]	0.5	*M*_0 _ *ra*_ mmHg [15]	0.5
*V*_0 _ *lv*_ ml [15]	25	*V*_0 _ *rv*_ ml [15]	25
*V*_0 _ *la*_ ml [15]	20	*V*_0 _ *ra*_ ml [15]	20
*V*_*d* _ *lv*_ ml [15]	40	*V*_*d* _ *rv*_ ml [15]	40
*V*_*d* _ *la*_ ml [15]	20	*V*_*d* _ *ra*_ ml [15]	20
*λ*_*lv*_ ml^−1^[15]	0.015	*λ*_*rv*_ ml^−1^[15]	0.015
*λ*_*la*_ ml^−1^[15]	0.025	*λ*_*ra*_ ml^−1^[15]	0.025

**Table 3:** Parameters of the activation functions [14, 15]

Parameter	*e*_*la*_(*t*)/*e*_*ra*_(*t*)	*e*_*lv*_(*t*)/*e*_*rv*_(*t*)			
	*i* = 0	*i* = 1	*i* = 2	*i* = 3	*i* = 4
*A*_*i*_ (left, right)	0.9	0.3	0.35	0.5	0.55
*B*_*i*_ (left, right)	0.038	0.045	0.035	0.037	0.036
*C*_*i*_ (left)	0.145	0.275	0.33	0.375	0.4
*C*_*i*_ (right)	0.125	0.288	0.343	0.388	0.413

### Nonlinear P-V Relations for Specified Vessels

The P-V relations of systemic veins, superior and inferior vena cava, and proximal systemic artery are non-linear, and the compliance of these vessels varies with pressure and volume. The nonlinear vascular model was proposed by Lu and Clark et al. [14], in which the compliances were expressed by P-V relation, meanwhile the vascular resistances of the superior, inferior vena cava, and proximal systemic artery were nonlinear functions of blood volume.

#### Systemic Veins

The physiological knowledge tells that, compared to artery, vein has thin and soft wall. The diameter is usually greater than that of artery. The wall of vein usually collapses in normal condition. Therefore, veins have small elasticity accordingly, like blood containers. At the beginning of increasing volume, the vein deformation is almost inconspicuous. However, with increasing volume, the vein undergoes a large deformation, which causes the venous pressure to rise quickly. Therefore, the veins stiffen as blood volume increases, whose P-V relation is nonlinearly modeled as [14],

7 $$ {P}_{sv}\left({V}_{sv}\right)=-{K}_v\times {\log}_{10}\left(\frac{V_{sv,\max }}{V_{sv}}-0.99\right), $$

where *P*_*sv*_ and *V*_*sv*_ are the pressure and volume of systemic veins, respectively. *K*_*v*_ is the scaling factor, and *V*_*sv*, max_ is the maximum volume of systemic veins. In normal condition, *V*_*sv*_ is about 2610 ml, *P*_*sv*_ is about 17~18 mmHg.

#### Vena Cava

The P-V relation of the vena cava is a stepwise function [14],

8 $$ {P}_{vc}\left({V}_{vc}\right)=\Big\{{\displaystyle \begin{array}{c}{N}_1+{K}_1\times \left({V}_{vc}-{V}_{vc,0}\right),\\ {}{N}_2+{K}_2\times {e}^{V_{vc}/{V}_{vc,\min }},\end{array}}{\displaystyle \begin{array}{c}{V}_{vc}\ge {V}_{vc,0}\\ {}{V}_{vc}<{V}_{vc,0}\end{array}}, $$

where *P*_*vc*_ and *V*_*vc*_ are the pressure and volume of vena cava. *V*_*vc*, 0_ and *V*_*vc*, min_ are the unstressed and minimum volume, respectively. The P-V relation is able to simulate the human venous system by adjusting the parameters of *K*_1_, *K*_2_, *N*_1_, and *N*_2_. The resistance of the vena cava is [14]

9 $$ {R}_{vc}\left({V}_{vc}\right)={K}_R\times \frac{V_{vc,\max }}{V_{vc}}+{R}_0, $$

where *K*_*R*_ is the scaling factor, *R*_0_ is the offset parameter, and *V*_*vc*, max_ denotes the maximum volume.

#### Proximal Systemic Artery

The compliance and resistance of proximal systemic artery are related to vasoconstriction, which is controlled by normalized sympathetic efferent frequency, *F*_*vaso*_. Hence, the P-V relation for proximal systemic artery is represented by both fully activated and passive states [14],

10a $$ {P}_{sap}^a\left({V}_{sap}\right)={K}_c\times {\log}_{10}\left(\frac{V_{sap}-{V}_{sap,\min }}{N_0}+1\right), $$

10b $$ {P}_{sap}^p\left({V}_{sap}\right)={K}_{p1}\times {e}^{\tau_{aop}\times \left({V}_{sap}-{V}_{sap,\min}\right)}+{K}_{p2}\times {\left({V}_{sap}-{V}_{sap,\min}\right)}^2, $$

10c $$ {P}_{sap}\left({V}_{sap}\right)={F}_{vaso}\times {P}_{sap}^a\left({V}_{sap}\right)+\left(1-{F}_{vaso}\right)\times {P}_{sap}^p\left({V}_{sap}\right), $$

where ${P}_{sap}^a$ and ${P}_{sap}^p$ are the pressures of proximal systemic artery in the fully activated and passive pressures, respectively. *V*_*sap*_ is the volume, and *V*_*sap*, min_ is the minimum volume. *K*_*c*_, *K*_*p*1_, and *K*_*p*2_ are the scaling factors. *N*_0_ is a volume parameter and *τ*_*aop*_ is a constant. The resistance of the proximal systemic artery is [14]

11 $$ {R}_{sap}\left({V}_{sap}\right)={K}_r\times \left[{e}^{4\times {F}_{vaso}}+{\left(\frac{V_{sap,\max }}{V_{sap}}\right)}^2\right], $$

where *K*_*r*_ is the scaling factor and *V*_*sap*, max_ is the maximal volume. All the parameters of the model are shown in Table 4.

**Table 4:** Parameters for nonlinear P-V relations of specified vessels [14, 15]

Parameter	Value	Parameter	Value
Systemic veins		*V*_*vc*, max_ ml	350
*K*_*v*_ mmHg	40	*V*_*vc*, min_ ml	50
*V*_*sv*, max_ ml	3500	Proximal systemic artery	
Vena cava		*N*_0_ ml	50
*N*_1_ mmHg	0	*K*_*c*_ mmHg	1000
*N*_2_ mmHg	−5	*K*_*p*1_ mmHg	0.03
*K*_1_ mmHg	0.15	*K*_*p*2_ mmHg ml^−2^	0.2
*K*_2_ mmHg	0.4	*K*_*r*_ mmHg s ml^−1^	0.04
*K*_*R*_ mmHg s ml^−1^	0.001	*V*_*sap*, *min*_ ml	210
*R*_0_ mmHg s ml^−1^	0.025	*V*_*sap*, max_ ml	250
*V*_*vc*, 0_ ml	130	*τ*_*aop*_ ml^−1^	0.1

#### Linear P-V Relations for General Vessels

Besides the specified vessels mentioned above, the P-V relations of other vessels, such as proximal pulmonary arteries, distal pulmonary arteries, and pulmonary veins, are modeled as linearity if there is no special explanation,

12 $$ P(t)=V(t)/C. $$

For example, based on this relation, *P*_lpap_(*t*) = *V*_lpap_(*t*)/*C*_lpap_, *P*_rpap_(*t*) = *V*_rpap_(*t*)/*C*_rpap_, etc. That is, in normal conditions, the compliances of these vessels are directly related to the value of *C*.

### Heart Rate Controls of the Model

The heart rate is controlled by vagal and sympathetic neural activity that is described as a three-dimensional response by Sunagawa [16]. The human heart rate response is further improved by Lu and Clark [14],

13 $$ Hr={h}_1+{h}_2\times {F}_{Hrs}-{h}_3\times {F}_{Hrs}^2-{h}_4\times {F}_{Hrv}+{h}_5\times {F}_{Hrv}^2-{h}_6\times {F}_{Hrv}\times {F}_{Hrs}, $$

where *h*_1_~*h*_6_ are the constants shown in Table 5, *F*_*Hrs*_ and *F*_*Hrv*_ represent normalized sympathetic and vagal frequencies, respectively.

**Table 5:** Parameters for control of heart rate [14, 15]

Heart rate					
*h*_1_	*h*_2_	*h*_3_	*h*_4_	*h*_5_	*h*_6_
35	140	40	32	10	20

### Solution to the Blood Circulation Model

The relations between compliance *C*, inductance *L*, blood flow *Q*(*t*), and blood pressure *P*(*t*) in the circuit system are

14a $$ Q(t)=C\frac{dP(t)}{dt}, $$

14b $$ P(t)=L\frac{dQ(t)}{dt}. $$

By using the relation between pressure and volume for the compliance, *V*(*t*) = *C* ⋅ *P*(*t*), Eqs. (14a, 14b) can be written as

15a $$ {V}^{\prime }(t)=\frac{dV(t)}{dt}=Q(t), $$

15b $$ {Q}^{\prime }(t)=\frac{dQ(t)}{dt}=\frac{P(t)}{L}. $$

Therefore, the platform shown in Fig. 1 can be transformed into a group of differential equations. The blood pressure and flow at any node of the platform can be numerically calculated.

### Simulated Normal Hemodynamics

**Fig. 2:**
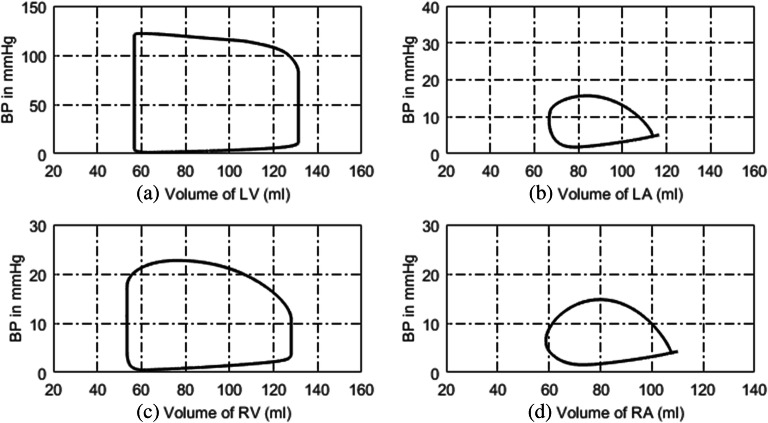
P-V loops of four heart chambers for normal state. **a** P-V loop of left ventricle. **b** P-V loop of left atrium. **c** P-V loop of right ventricle. **d** P-V loop of right atrium

**Fig. 3:**
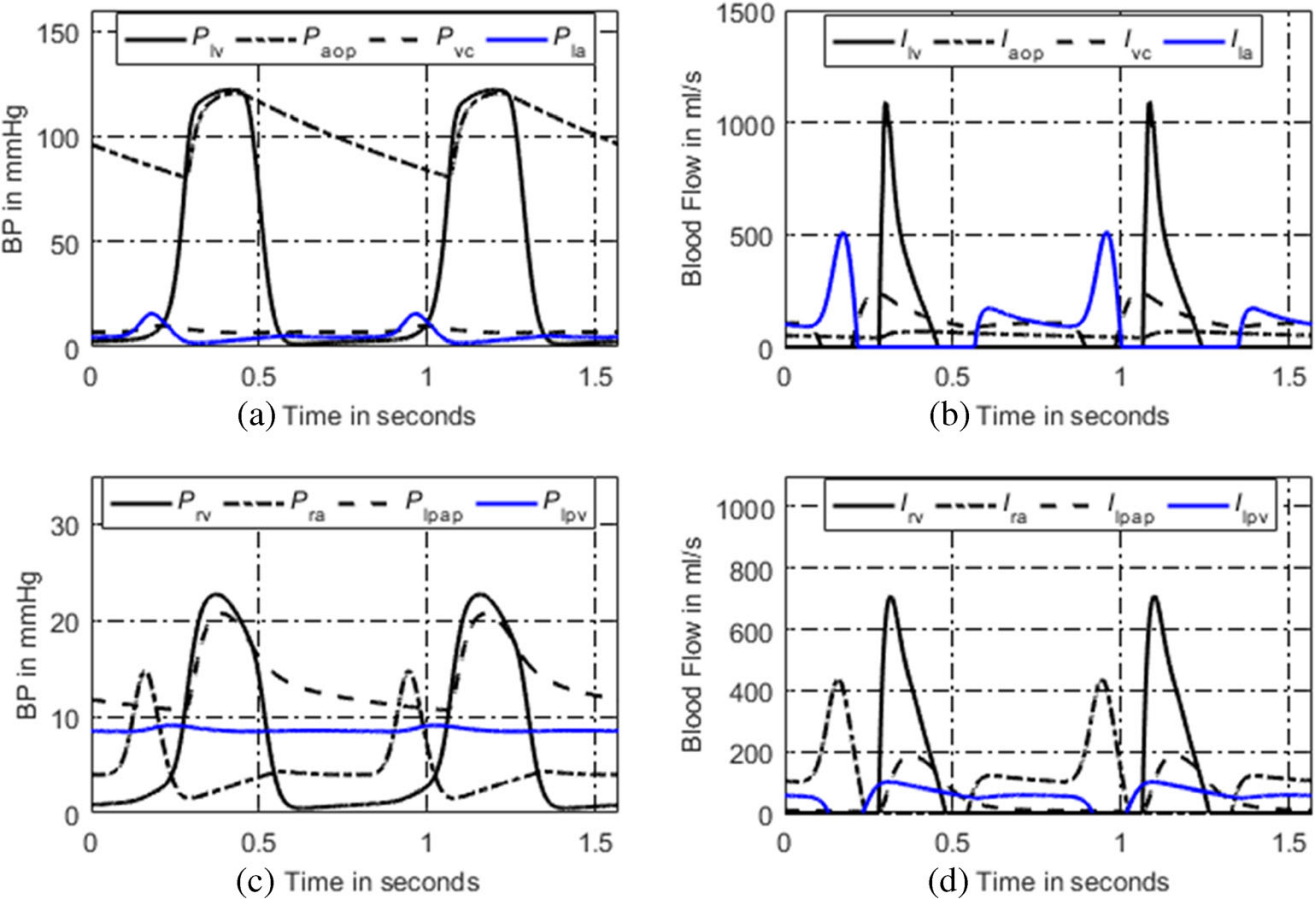
Simulated hemodynamics of two cardiac cycles. **a** Key systemic blood pressures. **b** Corresponding blood flows of **a**. **c** Key pulmonary blood pressures. **d** Corresponding pulmonary blood flows of **c**. Note for abbreviations, P: blood pressure; I: blood flow; lv: left ventricle; aop: proximal aorta; vc: vena cava; la: left atrium; rv: right ventricle; ra: right atrium; lpap: left proximal pulmonary artery; lpv: left pulmonary veins

The simulated P-V loops of four heart chambers for normal hemodynamics are shown in Fig. 2a–d. The blood pressure, blood flow at some key systemic, and pulmonary nodes are shown in Fig. 3a–d. It can be seen that the lumped-parameter circuit platform works like a normal human circulation system. The left ventricle pumps blood into aortic artery with systolic pressure 122 mmHg. The aortic artery receives blood and pushes blood forward where the pressure varies from 80 to 120 mmHg. The instantaneous flow at the outlet of left ventricle is seen in Fig. 3b. At the end of systemic circulation, the pressure in systemic vein is down to almost zero and has little variation. The pulmonary related pressures and flows at representative nodes and branches are illustrated in Figs. 2c, d and 3c, d. They all show that the simulated circulation system works in a normal state.

## Simulations for Four Typical Cases of PH

The circuit network shown in Fig. 1 can be used as a platform for simulating PH. So, a case of PH would occur and develop if a cause is imposed in the platform. Heart chambers and great vessels regulate their functions following special laws, accordingly. The underlying causes of PH are mechanical compression, distortion of the resistance vessels, vasoconstriction, disorders of the left side of the heart, and congenital heart disease [17, 18]. Though PH cases are different, there are many similar laws. Table 6 is a comparison table for presented cases which lists model commonalities and differences. This table may navigate reading.

**Table 6:** Navigation for presented cases. The numbers in the table are the equation index

Component name	Normal	DPAS	LVDD	VSD	MS
Left atrium	Atrial model	–	(38)~(40) (41)~(42)	(38) (44)~(45)	(47)~(49) (50)~(51)
Left ventricle	Ventricular model	–	(29)–(31)	–	–
Right atrium	Atrial model	–	–	–	–
Right ventricle	Ventricular model	(28)	(37)	(37)	(37)
Valves	Diode + resistance	–	–	–	Resistance of mitral valve (46)
Ventricular septal	–	–	–	A branch (43)	–
Sys. arteries	(10)~(11)	–	–	–	–
Sys. veins	(7)~(9)	–	–	–	–
Proximal Pul. arteries	(12)	(27)	(32a) (32b) (33a) (33b) (34) (35a) (35b) (36a) (36b)	(32a) (32b) (33a) (33b) (34) (35a) (35b) (36a) (36b)	(32a) (32b) (33a) (33b) (34) (35a) (35b) (36a) (36b)
Distal Pul. arteries	(12)	(23)	(32c) (32d) (33c) (33d) (34) (35c) (35d) (36c) (36d)	(32c) (32d) (33c) (33d) (34) (35c) (35d) (36c) (36d)	(32c) (32d) (33c) (33d) (34) (35c) (35d) (36c) (36d)
Pul. veins	(12)	(12)	(32e) (32f) (33e) (33f) (34) (35e) (35f) (36e) (36f)	(32e) (32f) (33e) (33f) (34) (35e) (35f) (36e) (36f)	(32e) (32f) (33e) (33f) (34) (35e) (35f) (36e) (36f)

Note: “–” no change

### PH Due to Distal Pulmonary Artery Stenosis

**Fig. 4:**
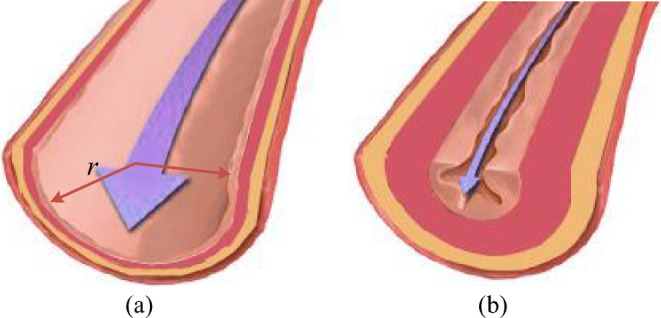
Schematic example of normal vessels and after vascular structure remodeling. **a** Healthy pulmonary artery. **b** Thick and stiff pulmonary artery

If pulmonary arteries are healthy and flexible, blood runs easily through the vessels. The synergistic effects of vasoconstriction, pulmonary vascular remodeling, and in-situ thrombosis cause an increase in pulmonary vascular resistance (PVR) and lead to PH. The increase in pulmonary artery pressure caused by pulmonary vasoconstriction is reversible in the early stage of PH. With the development of stenosis, the intima and medial membrane thickens, resulting in thickening of the vessel wall, narrowing of the lumen and remodeling of angiogenesis, which show irreversible changes in vascular structure. Thick and stiff artery walls limit blood flow and increase the resistance. As the artery narrows further, blood flow is restricted. Pulmonary vascular remodeling is the main pathological change of PH. The change of vascular radius before and after vascular remodeling is shown in Fig. 4.

#### Model of Nonlinear P-V Relation for Distal Pulmonary Arteries Due to Stenosis

Based on the well-known Poiseuilli’s law, the flow of liquid *Q* is proportional to the pressure difference at both ends of the pipe and the fourth order of the pipe radius, and inversely proportional to the length of the pipe,

16 $$ Q=\pi \left(\varDelta P\right)\;{r}^4/8\eta L, $$

where *r* is the radius of the pile, *ΔP* is the pressure difference, *L* is the pipe length, and *η* is the liquid viscosity. The blood flow resistance *R* is similar to the charge flow in a conductor, which is not directly measurable. *Q* is proportional to *ΔP* and inversely proportional to *R* which is known by using Ohm’s law,

17 $$ Q=\varDelta P/R, $$

thus the blood flow resistance, *R*, is inversely proportional to the fourth power of *r*,

18 $$ R=8\eta L/\pi {r}^4. $$

Assume *L* and *η* are constants. In order to simulate the development of distal pulmonary arteries narrowing over time, the radius decreases as a function of time, that is

19 $$ r(t)={r}_0{\left(1+{g}_r\times t\right)}^{-1/4}, $$

where *r*_0_ is the initial radius and *g*_*r*_ is used for changing rate. Clinical observations indicate that the resistance develops slowly and the progress may take years [19]. So, the time variable *t* in Eq. (19) and later is defined at large time scale. It is reasonable to assume that the artery suffering stenosis has no change in short time. Then, the artery could be in a steady state in short time and Poiseuille law is valid, accordingly. The short time in this study is supposed to be a single cardiac cycle duration. Therefore, the artery could be believed having no change in a cardiac cycle. This study simulates the stenosis of the distal left, right pulmonary arteries *R*_*rpad*_ and *R*_*lpad*_ by this way. So, the relations between *R*_*rpad*_, *R*_*lpad*_, and *r* can be written as

20a $$ {R}_{rpad}\left(r(t),t\right)={\left(1/r(t)\right)}^4\times {R}_{rpad,0}, $$

20b $$ {R}_{lpad}\left(r(t),t\right)={\left(1/r(t)\right)}^4\times {R}_{lpad,0}, $$

where *R*_*lpad*, 0_ and *R*_*rpad*, 0_ are the initial values of *R*_*lpad*_ and *R*_*rpad*_ which are given in Table 7. These initial values are calculated from the platform in normal condition (resistance equals pressure difference divided by blood flow). Clinical data in [20] showed that the radius of pulmonary artery could reduce 50%. Then, the resistance may become 1/(0.5)^4^ = 16 times of the initial value.

**Table 7:** Initial values of pulmonary vascular resistances in the pulmonary circulation

Parameter	Value	Parameter	Value
*R*_*rpap*, 0_	0.05 mmHg s ml^−1^	*R*_*lpap*, 0_	0.05 mmHg s ml^−1^
*R*_*rpad*, 0_	0.06 mmHg s ml^−1^	*R*_*lpad*, 0_	0.06 mmHg s ml^−1^
*R*_*rpv*, 0_	0.07 mmHg s ml^−1^	*R*_*lpv*, 0_	0.07 mmHg s ml^−1^

The previous studies [21, 22] showed that the resistance *R* and compliance *C* are inversely related. However, recent emerging evidence suggests that this concept should be challenged [23], their product decreases as normalized pulmonary vascular stiffness increases. This study accepts the new conclusion that product of *R* and *C*, called the *RC*-time, decreases over time,

21a $$ R\times C=\tau (t), $$

21b $$ \tau (t)={\tau}_0\cdot {e}^{-\sigma t}. $$

In this paper, *τ*_0_ is the initial value of *RC*-time in the normal heart, and *σ* is a parameter to control the change rate. Hence, the compliance of distal left and right pulmonary arteries *C*_*rpad*_ and *C*_*lpad*_ are

22a $$ {C}_{rpad}\left(r(t),t\right)=\tau (t)\cdot r{(t)}^4/{R}_{rpad,0}, $$

22b $$ {C}_{lpad}\left(r(t),t\right)=\tau (t)\cdot r{(t)}^4/{R}_{lpad,0}. $$

Based on the relation between pressure and volume, *P*_*rpad*_ = *V*_*rpad*_/*C*_*rpad*_ and *P*_*lpad*_ = *V*_*lpad*_/*C*_*lpad*_, hence, the P-V relations of the distal right, left pulmonary arteries implied by Eqs. (22a) and (22b) are obtained by integration as

23a $$ {P}_{rpad}\left({V}_{rpad},r(t),t\right)=\frac{V_{rpad}\cdot {R}_{rpad,0}}{\tau (t)\cdot {r}^4(t)}, $$

23b $$ {P}_{lpad}\left({V}_{lpad},r(t),t\right)=\frac{V_{lpad}\cdot {R}_{lpad,0}}{\tau (t)\cdot {r}^4(t)}. $$

#### Nonlinear P-V Relation for Proximal Pulmonary Arteries

With the development of PH, the pressures in proximal left and right pulmonary arteries gradually increase to abnormal high state. The P-V relation becomes nonlinear to adapt the abnormality. On the basis of works proposed by Salazar et al. [24] and Hardy et al. [25], within physiological limits, the blood vessel is considered as a container for blood, in which increasing pressure causes an increasing vessel stiffness. *dV*/*dP* tends to zero as pressure *P* increases, and the volume *V* approaches the maximum volume *V*_*m*_. Therefore,

24 $$ k\cdot \left({V}_m-V\right)=\frac{dV}{dP}, $$

where *k* is the constant and *V*_*m*_ is the maximum value of the vessel volume. The pressure implied by Eq. (24) can be obtained by integration,

25 $$ P(V)={P}_a-\frac{1}{k}\ln \frac{V_m-V}{V_m-{V}_a}, $$

(*V*_*a*_, *P*_*a*_) is an arbitrary point on the P-V curve. The operator ln(⋅) is natural logarithm. If *V*_*a*_ = *P*_*a*_ = 0, it becomes,

26 $$ P(V)=-K\ln \left(1-V/{V}_m\right). $$

where *K* = 1/*k*. The nonlinear P-V relation is applied to the proximal left and right pulmonary arteries,

27a $$ {P}_{lpap}\left({V}_{lpap}\right)=-{K}_{lpap,0}\ln \left(1-{V}_{lpap}/{V}_{m, lpap}\right), $$

27b $$ {P}_{rpap}\left({V}_{rpap}\right)=-{K}_{rpap,0}\ln \left(1-{V}_{rpap}/{V}_{m, rpap}\right), $$

where *V*_*m*, *lpap*_ and *V*_*m*, *rpap*_ are the maximum volume of proximal left and right pulmonary arteries. *K*_*lpap*, 0_ and *K*_*rpap*, 0_ are the constants. The imaging technique has been set up to estimate the volume in normal and PH states, as many previous studies described. Then, combining the pressure in normal and PH states, it is easy to estimate *K*_*lpap*, 0_ and *K*_*rpap*, 0_. These parameters are given in section “Simulation Results of PH Caused by Distal Pulmonary Artery Stenosis”.

#### Compensation for Right Ventricular Contractibility.

Right ventricular systolic function is a comprehensive reflection of right ventricular contractility, afterload and preload. With the progress of distal pulmonary arteries stenosis, PVR, afterload, and mean pulmonary artery pressure (mPAP) could gradually increase. In this case, right ventricular hypertrophy can be reformed by increasing the thickness and contractility of the ventricular wall in order to adapt to the continuous increase of mPAP. The compensation of right ventricle in this paper is achieved by increasing right ventricular end-systolic elastance, *E*_*es* _ *rv*_.

The previous work [26] showed that *E*_*es* _ *rv*_ had an upward trend with the aggravation of the disease, which increased rapidly in the early stage of PH, slowly in the middle and late stage. The maximum right ventricular elastance *E*_*es* _ *rv*, max_ is 1.30 ± 0.84 mmHg/ml, and mPAP = 1/3*sPAP+2/3*dPAP, where sPAP and dPAP are the systolic and diastolic blood pressure in the proximal pulmonary arteries. In this paper, *E*_*es* _ *rv*_ is modeled to increase following a piecewise function over time,

28a $$ {E}_{es\_ rv}(t)={E}_{es\_ rv,0}+{k}_1\times t, mPAP<50 mmHg, $$

28b $$ {E}_{es\_ rv}(t)={E}_{es\_ rv,0}+{k}_1\times {t}_c+{k}_2\times \left(t-{t}_c\right), mPAP\ge 50 mmHg. $$

The piecewise function has a breakpoint as the mPAP reaches 50 mmHg, and *t*_*c*_ is the time as*mPAP* ≥ 50*mmHg*, *k*_1_ and *k*_2_ are the parameters to control the change rate. The simulation results for the occurring and development of PH caused by DPAS are shown in section “Simulation Results of PH Caused by Distal Pulmonary Artery Stenosis”.

### PH Caused by Left Ventricular Diastolic Dysfunction

**Fig. 5:**
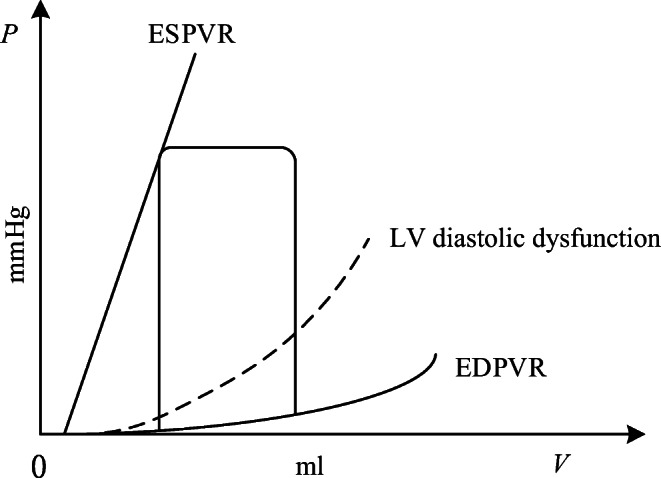
Schematic diagram of P-V relation for normal and left ventricular diastolic dysfunction. Dashed line indicates EDPVR in the left ventricular diastolic dysfunction

LVDD is one of the most common causes to lead to PH. The decrease of left ventricular myocardial compliance and filling disorder result in excessive left ventricular end-diastolic pressure, which increase left atrial filling pressure. Because of this dysfunction, the left heart is unable to keep up with blood returning from the lungs. Pressure in the lungs raises, pulmonary hypertension and congestion occur consequently [27]. The previous study showed that the contractile function of the myocardium had no change and the ESPVR was the same as a normal heart in the LVDD, but the P-V relation during diastole shifted upwards as shown in Fig. 5, meanwhile the ejection fraction (EF) was normal or slightly decreased [28].

**Fig. 6:**
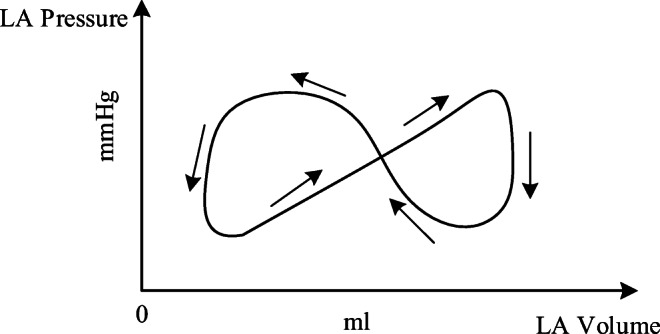
Schematic diagram of P-V relation of left atrium in PH caused by LVDD

In LVDD development, impaired left ventricular relaxation and increased passive stiffness is the principal functional derangement [28, 29]. Because of increasing in left ventricular end-diastolic pressure, there is an increase in left atrial and pulmonary venous pressure. Hence, pulmonary artery pressure increases consequently. As shown in previous study, left atrial structure and function were altered by increased LA stiffening and greater LA pressure [30, 31]; meanwhile, left atrial remodeling occurred in patients with LVDD, and LA volume expressed the severity of diastolic dysfunction [32, 33]. The P-V loop of LA is out of normal relation and shifts to a trend characterized by two loops. This relation differs greatly to that of a normal left atrium, see in Fig. 6. In addition, the compliances of pulmonary arteries have also changed due to the accumulation of blood in the pulmonary circulation. In the early stage of this PH, there may be no significant change in PVR. However, as the disease progresses, it eventually damages the pulmonary blood vessels, resulting in an increase in PVR [34].

This PH is closely related to the right heart. The concept of integration of right ventricle and pulmonary circulation has been proposed by researchers previously [35]. In normal physiological conditions, the right ventricle is connected to the low pressure, low resistance, and high compliance pulmonary circulation, and the right ventricle is sensitive to increased pressure load. In the early stage of PH, the right ventricle will compensate for the increase of pulmonary artery pressure. With the development of the disease, in order to adapt to the continuous increase of afterload and maintain the ability of ejection, right ventricle becomes hypertrophy until right heart failure occurs finally. Previous researchers have attempted to compensate for right ventricle [23]. In this paper, the compensation of right ventricle is achieved by increasing right ventricular end-systolic elastance, *E*_*es* _ *rv*_.

#### Model of EDPVR for Left Ventricle.

The P-V loop of left ventricle is the most direct manifestation of hemodynamic abnormalities. As shown in Fig. 5, the EDPVR shifts upwards in the LVDD [36], which is an exponential function controlled by *M*_*lv*_ and *λ*_*lv*_. In order to simulate the pathogenesis of LVDD, it is necessary to increase the values of *M*_*lv*_ and *λ*_*lv*_ with respect to time to raise the left ventricle diastolic pressure,

29 $$ {M}_{lv}(t)={M}_{lv,0}+{k}_3\times t, $$

30 $$ {\lambda}_{lv}(t)={\lambda}_{lv,0}+{k}_4\times t, $$

where *k*_3_ and *k*_4_ are the coefficients, *M*_*lv*, 0_ and *λ*_*lv*, 0_ are the initial values of *M*_*lv*_ and *λ*_*lv*_. That is, to simulate PH development of this case, the EDPVR relation shown in Eq. (2b) becomes

31 $$ {P}_{lv\_ ED}\left({V}_{lv},{M}_{lv}(t),{\lambda}_{lv}(t),t\right)={M}_{lv}(t)\times \mid \exp \left[{\lambda}_{lv}(t)\left({V}_{lv}-{V}_{0\_ lv}\right)\right]-1\mid . $$

#### Model of P-V Relation for Pulmonary Vessels

In the development of LVDD, the authors assume that the compliance of the blood vessels in the pulmonary circulation varies within a reasonable range. The P-V relations of proximal and distal pulmonary arteries and pulmonary veins are given in Eq. (26). The end-diastolic pressure is increased due to LVDD, causing obstruction of left atrial and pulmonary veins. Therefore, blood is deposited in the left atrium and pulmonary circulatory system, which in turn affects vessel elasticity in the pulmonary circulation. In the process of increasing blood accumulation, the parameters *K*(*t*) for proximal right and left pulmonary arteries, distal right and left pulmonary arteries, right and left pulmonary veins increase over time and vary within a reasonable range, which are given as,

32a $$ {K}_{rpap}(t)={K}_{rpap,0}+{k}_5\times t, $$

32b $$ {K}_{lpap}(t)={K}_{lpap,0}+{k}_5\times t, $$

32c $$ {K}_{rpad}(t)={K}_{rpad,0}+{k}_6\times t, $$

32d $$ {K}_{lpad}(t)={K}_{lpad,0}+{k}_6\times t, $$

32e $$ {K}_{rpv}(t)={K}_{rpv,0}+{k}_7\times t, $$

32f $$ {K}_{lpv}(t)={K}_{lpv,0}+{k}_7\times t, $$

where *k*_5_, *k*_6_, and *k*_7_ are the coefficients to control change rate, *K*_*lpap*, 0_, *K*_*rpap*, 0_, *K*_*lpad*, 0_, *K*_*rpad*, 0_, *K*_*lpv*, 0_, and *K*_*rpv*, 0_ are the constants. Based on clinical examination, the blood volume in the pulmonary circulation is about 450 ml. The average blood volume of left and right pulmonary veins is about 100 ml [37]. The normal blood volume in proximal right and left pulmonary arteries, distal right and left pulmonary arteries, and right and left pulmonary veins are approximately estimated as 50 ml, 70 ml, and 100 ml. The associated normal pressure therein could be 13 mmHg, 9 mmHg, and 5 mmHg. Previous study [38] showed that the essential cause of passive PH was excessive blood volume in the second type of PH. The references [39, 40] also showed that the pulmonary blood volume variation was higher in patients compared to healthy controls. The corresponding blood volumes in this PH case could be 80 ml, 110 ml, and 140 ml, which are less than twice of those normal. The associated pressure therein could be 90 mmHg, 65 mmHg, and 55 mmHg. So, *K*(*t*) could be determined accordingly in reasonable ranges, i.e., 19<*K*_*rpap*_(*t*)<56, 19<*K*_*lpap*_(*t*)<56, 14<*K*_*rpad*_(*t*)<49, 14<*K*_*lpad*_(*t*)<49, 4<*K*_*rpv*_(*t*)<37, and 4<*K*_*lpv*_(*t*)<37. The P-V relations for pulmonary vessels become

33a $$ {P}_{rpap}\left({V}_{rpap},{K}_{rpap}(t),t\right)=-{K}_{rpap}(t)\times \ln \left(1-{V}_{rpap}/{V}_{m, rpap}\right), $$

33b $$ {P}_{lpap}\left({V}_{lpap},{K}_{lpap}(t),t\right)=-{K}_{lpap}(t)\times \ln \left(1-{V}_{lpap}/{V}_{m, lpap}\right), $$

33c $$ {P}_{rpad}\left({V}_{rpad},{K}_{rpad}(t),t\right)=-{K}_{rpad}(t)\times \ln \left(1-{V}_{rpad}/{V}_{m, rpad}\right), $$

33d $$ {P}_{lpad}\left({V}_{lpad},{K}_{lpad}(t),t\right)=-{K}_{lpad}(t)\times \ln \left(1-{V}_{lpad}/{V}_{m, lpad}\right), $$

33e $$ {P}_{rpv}\left({V}_{rpv},{K}_{rpv}(t),t\right)=-{K}_{rpv}(t)\times \ln \left(1-{V}_{rpv}/{V}_{m, rpv}\right), $$

33f $$ {P}_{lpv}\left({V}_{lpv},{K}_{lpv}(t),t\right)=-{K}_{lpv}(t)\times \ln \left(1-{V}_{lpv}/{V}_{m, lpv}\right), $$

where *V*_*m*, *lpap*_, *V*_*m*, *rpap*_ *V*_*m*, *lpad*_, *V*_*m*, *rpad*_, *V*_*m*, *lpv*_, and *V*_*m*, *rpv*_ are the maximum volumes of corresponding vessels. The values of these parameters are given in section “Simulation Results of PH Caused by Left Ventricular Diastolic Dysfunction”.

#### Model of Pulmonary Vascular Resistances

For PH caused by LVDD, with the development of the disease, eventually irreversible damage to the pulmonary vessels results in increased PVR. Physiological knowledge tells that the vascular compliance will decrease with increasing pressure therein. The resistance will increase consequently. This has been observed by previous studies. Melenovsky et al. [33] observed that the PVR increased twice with increasing pressure in a PH case caused by heart failure. Raeisi-Giglou et al. [34] found from clinical observation that PVR became greater than normal in patients with LVDD. The previous study [22] showed that the mean pulmonary artery pressure *P*_*m*_ and compliance *C* show a relationship, which fit an exponential model,

34 $$ C\left({P}_m(t),t\right)={g}_c\cdot {e}^{-{h}_c\cdot {P}_m(t)}, $$

where *g*_*c*_ and *h*_*c*_ are the constant coefficients. Therefore, the resistances of the proximal right and left pulmonary arteries, distal right and left pulmonary arteries, and right and left pulmonary veins implied by Eqs. (21a), (21b), and (34) are obtained by integration as

35a $$ {R}_{rpap}\left({P}_{m\_ rpap}(t),t\right)=\frac{\tau_{rpap}(t)}{g_{cpl}\cdot {e}^{-{h}_{cpl}\cdot {P}_{m\_ rpap}(t)}}, $$

35b $$ {R}_{lpap}\left({P}_{m\_ lpap}(t),t\right)=\frac{\tau_{lpap}(t)}{g_{cpl}\cdot {e}^{-{h}_{cpl}\cdot {P}_{m\_ lpap}(t)}}, $$

35c $$ {R}_{rpad}\left({P}_{m\_ rpad}(t),t\right)=\frac{\tau_{rpad}(t)}{g_{cdl}\cdot {e}^{-{h}_{cdl}\cdot {P}_{m\_ rpad}(t)}}, $$

35d $$ {R}_{lpad}\left({P}_{m\_ lpad}(t),t\right)=\frac{\tau_{lpad}(t)}{g_{cdl}\cdot {e}^{-{h}_{cdl}\cdot {P}_{m\_ lpad}(t)}}, $$

35e $$ {R}_{rpv}\left({P}_{m\_ rpv}(t),t\right)=\frac{\tau_{rpv}(t)}{g_{cvl}\cdot {e}^{-{h}_{cvl}\cdot {P}_{m\_ rpv}(t)}}, $$

35f $$ {R}_{lpv}\left({P}_{m\_ lpv}(t),t\right)=\frac{\tau_{lpv}(t)}{g_{cvl}\cdot {e}^{-{h}_{cvl}\cdot {P}_{m\_ lpv}(t)}}, $$

where

36a $$ {\tau}_{rpap}(t)={\tau}_{rpap\_0}\cdot {e}^{-{\sigma}_{l\_ rpap}\cdot t}, $$

36b $$ {\tau}_{lpap}(t)={\tau}_{lpap\_0}\cdot {e}^{-{\sigma}_{l\_ lpap}\cdot t}, $$

36c $$ {\tau}_{rpad}(t)={\tau}_{rpad\_0}\cdot {e}^{-{\sigma}_{l\_ rpad}\cdot t}, $$

36d $$ {\tau}_{lpad}(t)={\tau}_{lpad\_0}\cdot {e}^{-{\sigma}_{l\_ lpad}\cdot t}, $$

36e $$ {\tau}_{rpv}(t)={\tau}_{rpv}\cdot {e}^{-{\sigma}_{l\_ rpv}\cdot t}, $$

36f $$ {\tau}_{lpv}(t)={\tau}_{lpv\_0}\cdot {e}^{-{\sigma}_{l\_ lpv}\cdot t}, $$

*g*_*cpl*_, *g*_*cdl*_, *g*_*cvl*_, *h*_*cpl*_, *h*_*cdl*_, and *h*_*cvl*_ are the constant coefficients. *τ*_*rpap* _ 0_, *τ*_*lpap* _ 0_, *τ*_*rpad* _ 0_, *τ*_*lpad* _ 0_, *τ*_*rpv* _ 0_, and *τ*_*lpv* _ 0_ are the initial values of *RC*-time for the proximal right and left pulmonary arteries and distal right, left pulmonary arteries, and right and left pulmonary veins in the normal heart. *σ*_*l* _ *rpap*_, *σ*_*l* _ *lpap*_, *σ*_*l* _ *rpad*_, *σ*_*l* _ *lpad*_, *σ*_*l* _ *rpv*_, and *σ*_*l* _ *lpv*_ are the parameters to control the changing rate in the case caused by LVDD.

#### Model for Right Ventricular Compensation

With the development of this type of PH, the right ventricle in the LVDD model overcomes the increase of afterload by increasing myocardial contractility *E*_*es* _ *rv*_ which is given as follows

37 $$ {E}_{es\_ rv}(t)={E}_{es\_ rv,0}+{k}_8\times t, $$

where *k*_8_ is the parameter to control change rate, *E*_*es* _ *rv*, 0_ is the initial value of *E*_*es* _ *rv*_.

#### Models for P-V Loop and Activation Function of Left Atrium

**Fig. 7:**
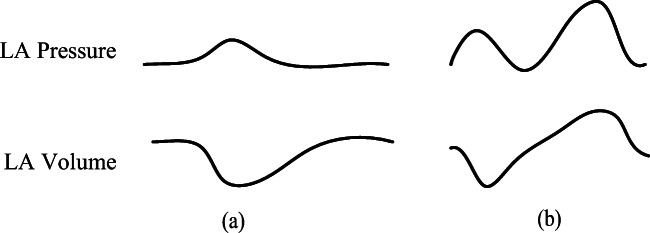
Relations between blood pressure and volume for left atrium. **a** For normal hemodynamics. **b** For abnormal hemodynamics due to LVDD

In the cardiovascular system, the left atrium acts as an elastic reservoir, passive conduit, and active booster to regulate left ventricular filling. Left atrium dysfunction and remodeling are common in patients with heart failure (HF). Increasing evidences showed that left atrial dysfunction was a positive cause of symptoms and disease progression [41]. In order to overcome the increase of left atrial pressure and volume caused by LVDD, previous studies on left atrial dysfunction disclosed that the P-V loop of the left atrium had changed [33, 34], as shown in Fig. 7. The systolic and diastolic blood pressures of LA are increased to adapt to the rise of left ventricular end-diastolic pressure and pulmonary vein pressure. Therefore, the parameters of *E*_*es* _ *la*_, *M*_*la*_, and *λ*_*la*_ in the left atrial model increase over time

38 $$ {E}_{es\_ la}(t)={E}_{es\_ la,0}+{k}_9\times t, $$

39 $$ {M}_{la}(t)={M}_{la,0}+{k}_{10}\times t, $$

40 $$ {\lambda}_{la}(t)={\lambda}_{la,0}+{k}_{11}\times t, $$

where *k*_9_, *k*_10_, and *k*_11_ are the parameters to control change rate, *E*_*es* _ *la*, 0_, *M*_*la*, 0_, and *λ*_*la*, 0_ are the initial values of *E*_*es* _ *la*_, *M*_*la*_, and *λ*_*la*_.

**Fig. 8:**
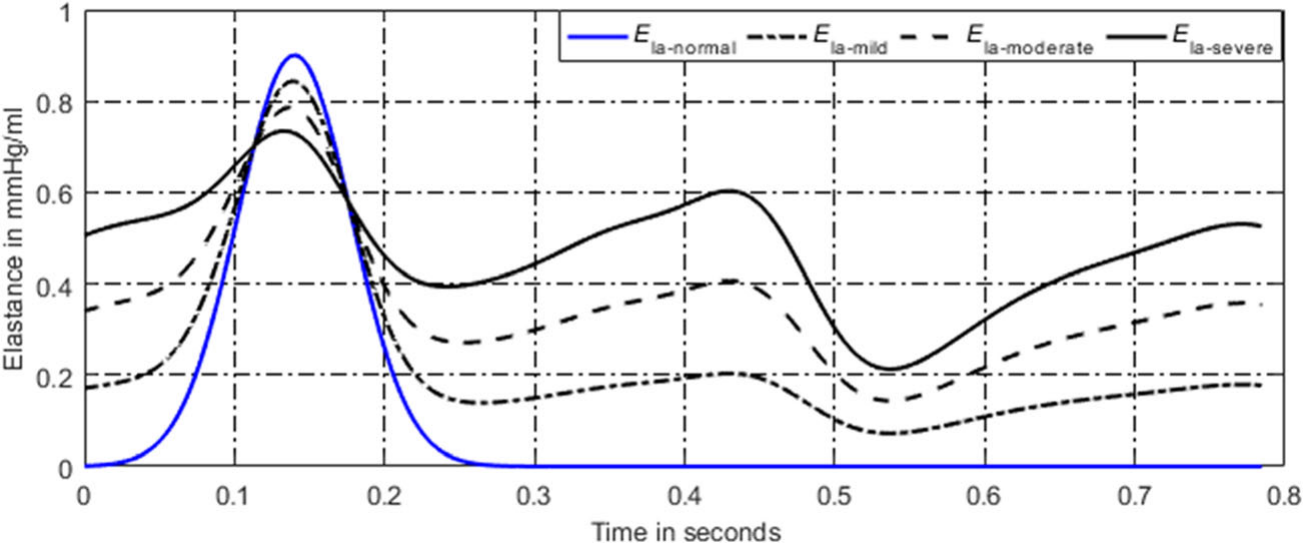
Regulation of left atrial activation function to adapt PH due to LVDD

**Fig. 9:**
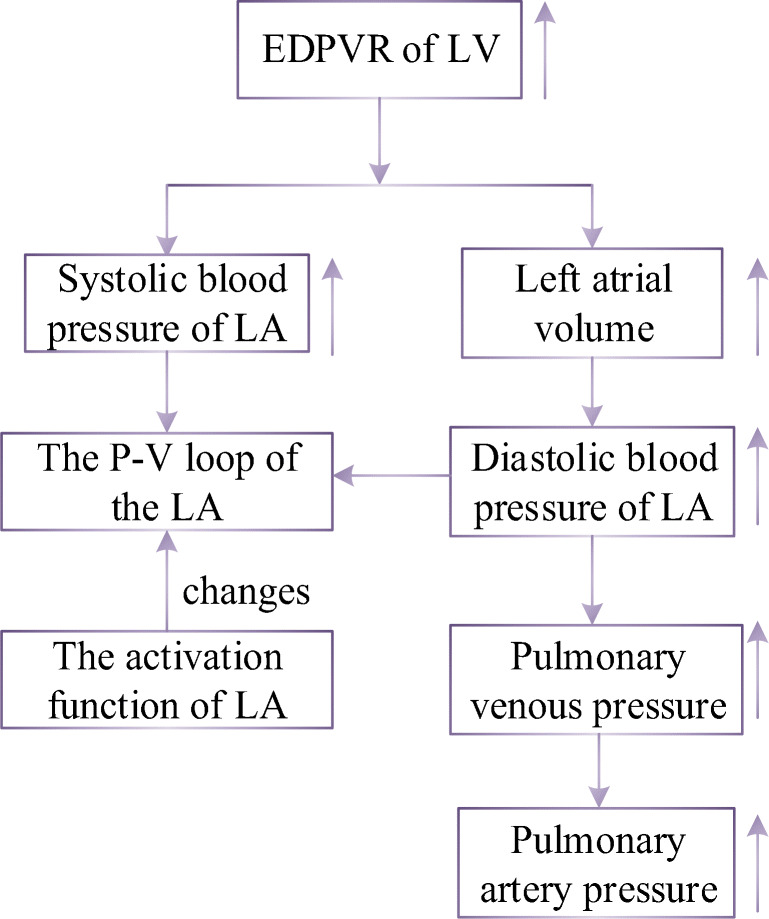
Pathophysiology of PH caused by LVDD

For a normal heart, the pressure and volume of left atrium in one cardiac cycle are shown in Fig. 7a. The activation function of the left atrium can be well modeled by one Gaussian function, see in Fig. 8. During the process from normal to this PH, left ventricular filling pressure continues to increase. The left atrium needs to raise systolic blood pressure continuously to push blood to the left ventricle. However, as the disease progresses, persistent long-term left ventricular end-diastolic pressure increases, which will cause block blood flow back to the left ventricle. Blood silts in the left atrium, leading to an increase in left atrial volume and diastolic blood pressure. Increased pressure retrogrades conduction to the pulmonary veins. Pressures in pulmonary veins and pulmonary artery increase in turn. The pathophysiology of PH due to left ventricular diastolic dysfunction is shown in Fig. 9. The varying pressure and volume of left atrium in one cardiac cycle are shown in Fig. 7b. Compared with the normal hemodynamic state, there are two peaks in the left atrial pressure, and the systolic and diastolic blood pressures are increased. Therefore, the authors propose a new left atrial activation function, see in Fig. 8, and the modified activation function is expressed by the sum of ten Gaussian functions,

41 $$ e{}_{la\_ LVDD}(t)=\sum \limits_{i=1}^{10}\left(a{}_i(t)\times \exp \left[-0.5\times {\left(\frac{{\left[t\right]}_T-{c}_i}{b_i}\right)}^2\right]\right). $$

As the disease progresses, the amplitude of the first peak decreases over time, and it becomes wide. The left atrial pressure increases during diastole; thus, the authors assume that the amplitude of Gaussian curves could vary in time with the following rules,

42 $$ {a}_i(t)={a}_{i,0}+{k}_{a,i}\times t,\kern1em i=1,\cdots, 10, $$

where *k*_*a*, *i*_ are the coefficients. The simulation results for the occurring and development of PH caused by LVDD are shown in section “Simulation Results of PH Caused by Left Ventricular Diastolic Dysfunction”.

### PH Caused by Ventricular Septal Defect

**Fig. 10:**
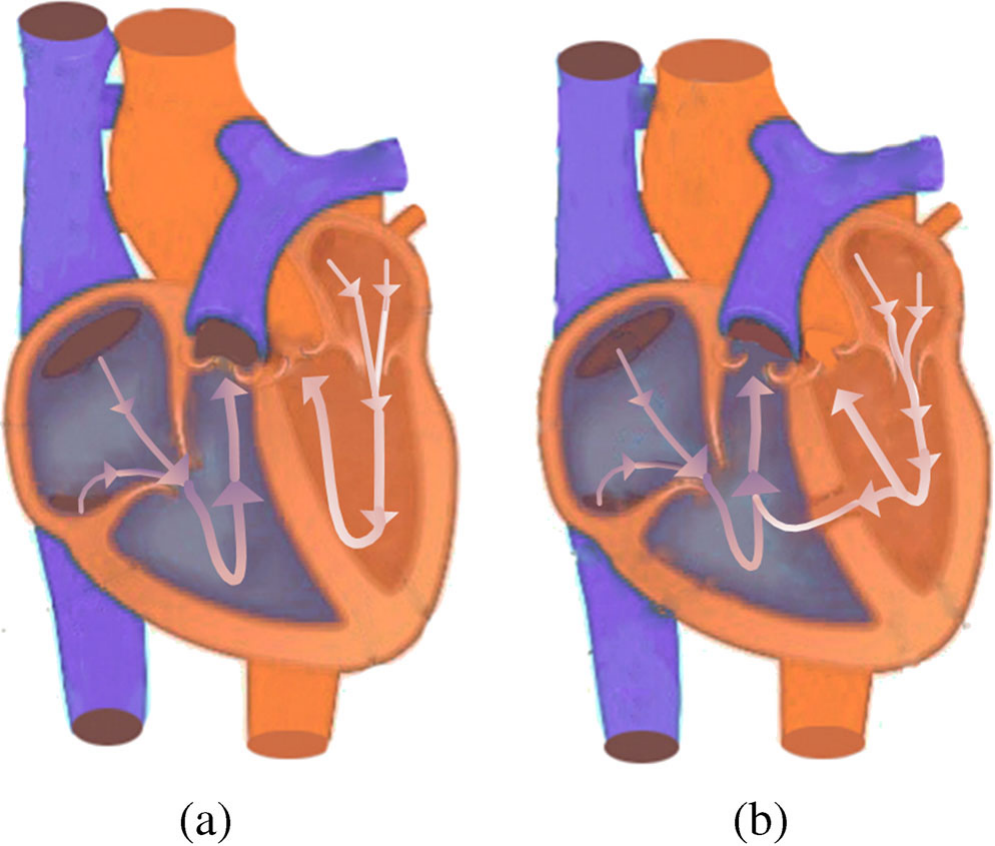
Illustration of blood flow for a normal heart and a heart with VSD. **a** Blood flow in normal heart. **b** Blood flow in heart with VSD

Congenital heart disease is one of the major causes of PH, and patients with VSD are the most common congenital cardiac disorder, characterized by an abnormal opening in the ventricular septum, which allows blood to shunt between the left and right ventricles [11, 42, 43], see in Fig. 10. Because the blood pressure of the left ventricle is much larger than that of the right ventricle, most of them originally are left-to-right shunting. The left-to-right shunting of congenital ventricular septal defect leads to an increase in pulmonary blood flow and pulmonary artery pressure, which in turn affects pulmonary vascular endothelial function, resulting in increased PVR, making to left-to-right shunting originally that develops bidirectional or right-to-left shunting, or appears cyanosis [44], that is, Eisenmenger syndrome (ES). ES is the terminal stage of PH in congenital heart disease, and about 50% of patients with VSD will eventually develop into ES.

PH caused by VSD also affects the function of right ventricle. The right ventricle needs to overcome the continuous increase of pulmonary artery pressure. As time goes on, the right ventricle will eventually decompensate, leading to right heart failure. Previous studies showed that in the early stage of PH caused by VSD, PVR could be normal, and only the pulmonary artery pressure increased. However, with the development of the disease, PVR would still increase, causing pulmonary vascular lesions to an irreversible stage [45, 46].

#### Model of Shunting Resistance

**Fig. 11:**
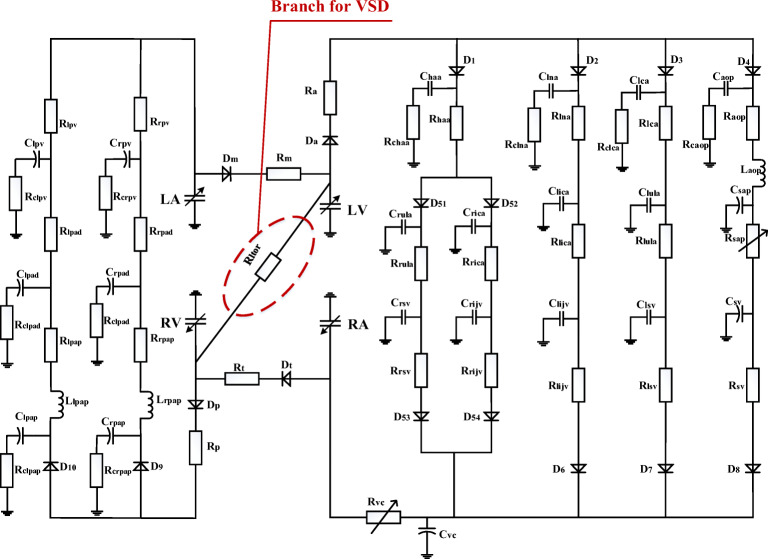
Analog circuit model for PH caused by VSD

VSD is characterized by an abnormal opening in the septum between the ventricles. Clinical investigation releases that the maximum defect area is possibly greater than 2 cm^2^ [47]. In this study, the open shunting is simulated as a branch using a resistor *R*_*ltor*_ in the circuit, see Fig. 11. In a normal heart, there is no blood flow through the septum. *R*_*ltor*_ is equivalent to an infinite resistance. From a physiological point of view, increasing opening area of VSD leads to increasing flow. That is to say, the value of resistance *R*_*ltor*_ is inverse to the opening area. The study in [11] observed that the resistance could be 1000 hydraulic resistance unit for normal and greatly reduced to 0.15 unit for large VSD. Authors are inspired by the nonlinear relation between mitral resistance and corresponding area, which was proposed by Beyer et al. [48]. If the opening area becomes larger and larger with VSD developing with respect to time, *R*_*ltor*_ could be simulated to decrease nonlinearly over time,

43 $$ {R}_{ltor}(t)={R}_{ltor,0}/{\left(1+{k}_r\times t\right)}^2, $$

where *R*_*ltor*, 0_ is the initial value of *R*_*ltor*_ in normal condition and *k*_*r*_ is the coefficient to control the change rate. Numerical simulation shows that the hemodynamic responses are very sensitive to *R*_*ltor*_ when *R*_*ltor*_ < 10 mmHg ⋅ s ⋅ ml^−1^, which provides knowledge on how to determine the *R*_*ltor*, 0_ and *k*_*r*_.

#### Model of Pulmonary Vascular Resistances.

Long-term left-to-right shunting VSD leads to increased blood flow in the pulmonary circulation, leads to abnormal pulmonary vascular endothelial function, and results in increased PVR. The previous study [22] showed that the vascular compliance decreased with increasing mean pulmonary artery pressure. The resistance would increase consequently. Increasing trend of resistances are applied to proximal right and left pulmonary arteries, distal right and left pulmonary arteries, and right and left pulmonary veins. The increasing laws are similar to those of Eqs. (34), (35a, 35b, 35c, 35d, 35e, 35f ), and (36a, 36b, 36c, 36d, 36e, 36f). The difference is that the coefficients for *g*_*cpv*_, *g*_*cdv*_, *g*_*cvv*_, *h*_*cpv*_, *h*_*cdv*_ and *h*_*cvv*_, and *σ*_*v* _ *rpap*_, *σ*_*v* _ *lpap*_, *σ*_*v* _ *rpad*_, *σ*_*v* _ *lpad*_, *σ*_*v* _ *rpv*_, *σ*_*v* _ *lpv*_ are parameters to control the changing rate in the case caused by VSD.

#### Model of Activation Function for Left Atrium

**Fig. 12:**
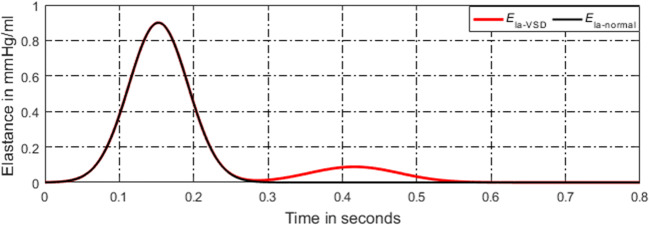
Regulation of left atrial activation function to adapt PH caused by VSD

In the development of VSD, a part of blood in the left ventricle flows into the right ventricle, directly involved in pulmonary circulation, leading to a large amount of blood entering the left atrium, resulting in abnormal enlargement of left atrial volume and increased pressure. In a normal heart, the left atrial activation function does not adapt to abnormal changes in pressure and volume of left atrium. Therefore, the authors propose a model of left atrial activation function to adapt the abnormal hemodynomics, see in Fig. 12, which is expressed by the sum of three Gaussian curves,

44 $$ e{}_{la\_ VSD}(t)=\sum \limits_{i=1}^3\left({\alpha}_i(t)\times \exp \left[-0.5\times {\left(\frac{{\left[t\right]}_T-{\omega}_i}{\beta_i}\right)}^2\right]\right), $$

As the disease progresses, both the systolic and diastolic blood pressures of left atrium increase. The proposed left atrial activation function has two peaks. The first peak is determined by the first Gaussian curve controlled by three constants *α*_1_(*t*), *ω*_1_, and *β*_1_. So, the first peak does not vary during the disease development, as seen in Fig. 12. However, the amplitude of the second peak, reflected by the second and the third Gaussian curves, increases over time,

45a $$ {\alpha}_1(t)={\alpha}_{1,0}, $$

45b $$ {\alpha}_2(t)={\alpha}_{2,0}+{k}_{\alpha_2}\times t, $$

45c $$ {\alpha}_3(t)={\alpha}_{3,0}+{k}_{\alpha_3}\times t, $$

where ${k}_{\alpha_2}$ and *k*_*α*__3_are the coefficients.

The P-V relations of proximal, distal pulmonary arteries, and pulmonary veins are given in Eqs. (33a, 33b, 33c, 33d, 33e, 33f), and the coefficients indicating increasing rates of the parameters *K*(*t*) are defined as *k*_*vsd* _ *p*_, *k*_*vsd* _ *d*_ and *k*_*vsd* _ *v*_, respectively. The compensation of right ventricle and left atrium are given by Eqs. (37)–(38), and the coefficients indicating increasing rates of the parameters *E*_*es* _ *rv*_(*t*) and *E*_*es* _ *la*_(*t*) are defined as *k*_*rv* _ *v*_ and *k*_*la* _ *v*_. The simulation results for the occurring and development of PH caused by VSD are shown in section “Simulation Results of PH Caused by Ventricular Septal Defect”.

### PH Caused by Mitral Stenosis

The mitral valve ensures the unidirectional flow of blood from left atrium to left ventricle. Pathological changes such as ischemic necrosis and trauma can cause abnormalities in the structure and function of the mitral valve, leading to MS. Under normal conditions, blood flowing from left atrium to left ventricle does not cause any obstacles. When the mitral stenosis occurs, the hemodynamics will obviously change [49, 50]. The blood flowing from left atrium to left ventricle encounters an obstacle, resulting in an increase in left atrial pressure, which in turn causes an increase in pressure in the pulmonary veins and pulmonary arteries, leading to PH. In addition, the right ventricle is in a long-term increase in post-load pressure, eventually resulting in right heart failure [51].

**Fig. 13:**
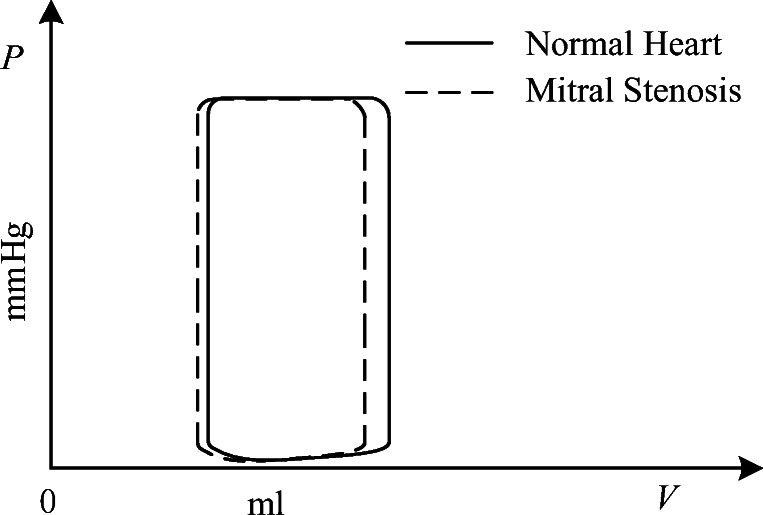
P-V relation of left ventricle caused by MS

Due to limited blood flowing from left atrium to left ventricle, the left ventricular end-diastolic volume and pressure are reduced, and left ventricular end-systolic volume and stroke volume are also decreased. The P-V relation of left ventricle under these conditions is shown in Fig. 13.

#### Model of Mitral Resistance.

In the analog circuit platform, the mitral valve is simulated by a resistor *R*_*m*_ and a diode *D*_*m*_. From a physiological point of view, the blood flow resistance of mitral valve increases gradually from opening to closing. Increasing degree of MS also contributes to the continuous increasing in *R*_*m*_. The increasing resistance *R*_*m*_ over time is modeled as,

46 $$ {R}_m(t)={R}_{m,0}+{k}_{12}\times t, $$

where *R*_*m*, 0_ is the initial value of *R*_*m*_, and *R*_*m*, 0_=0.02 mmHg·s·ml^−1^. And *k*_12_ is a parameter to control change rate.

#### 2) Model of Pulmonary Vascular Resistances

When the mitral stenosis occurs, the blood flowing from left atrium to left ventricle encounters an obstacle, resulting in an increase in left atrial pressure, which in turn causes an increase in pressure in the pulmonary veins and pulmonary arteries, and leads to PH. Previous study [22] showed that the vascular compliance decreased with increasing mean pulmonary artery pressure. The resistance would increase consequently. The increasing resistances are involved in pulmonary vessels, such as proximal right and left pulmonary arteries, distal right and left pulmonary arteries, and right and left pulmonary veins. The increasing laws are similar to those of Eqs. (34), (35a, 35b, 35c, 35d, 35e, 35f), and (36a, 36b, 36c, 36d, 36e, 36f). The difference is that the coefficients for *g*_*cpm*_, *g*_*cdm*_, *g*_*cvm*_, *h*_*cpm*_, *h*_*cdm*_ *h*_*cvm*_, and *σ*_*m* _ *rpap*_, *σ*_*m* _ *lpap*_, *σ*_*m* _ *rpad*_, *σ*_*m* _ *lpad*_, *σ*_*m* _ *rpv*_, *σ*_*m* _ *lpv*_ are parameters to control the changing rate in the case of a PH caused by MS.

#### Left Atrial Compensation for Contractibility.

MS is one of the left ventricular valve diseases. Pathological mechanisms may lead to elevated pressure in the left atrium. Therefore, the left atrium increases contractibility to overcome elevated pressure through its own regulation,

47 $$ {E}_{es\_ la}(t)={E}_{es\_ la,0}+{k}_{13}\times t, $$

48 $$ {M}_{la}(t)={M}_{la,0}+{k}_{14}\times t, $$

49 $$ {\lambda}_{la}(t)={\lambda}_{la,0}+{k}_{15}\times t, $$

where *k*_13_, *k*_14_, and *k*_15_ are the coefficients, *E*_*es* _ *la*, 0_, *M*_*la*, 0_, and *λ*_*la*, 0_ are the initial values of *E*_*es* _ *la*_, *M*_*la*_, and *λ*_*la*_.

#### Model of Activation Function for Left Atrium

**Fig. 14:**
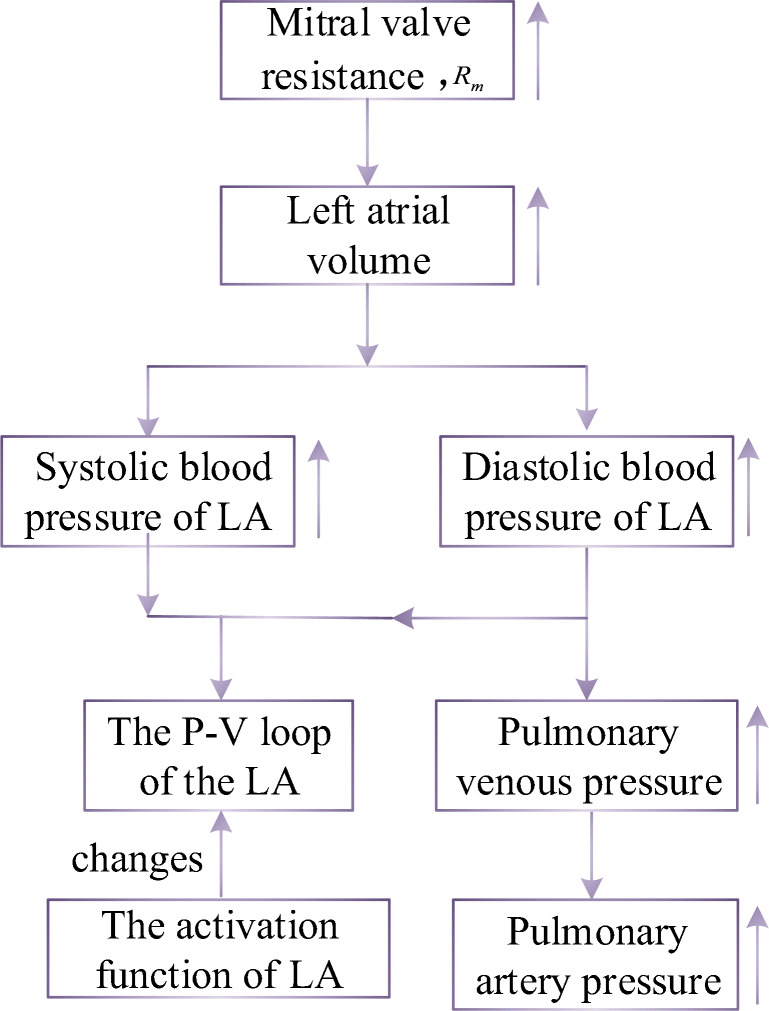
Pathophysiology of PH due to MS

**Fig. 15:**
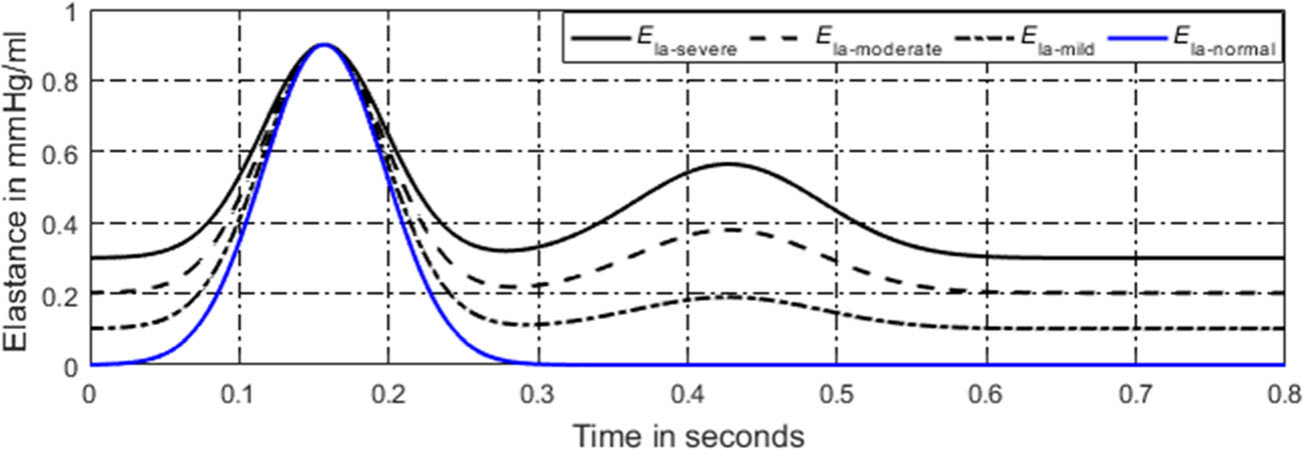
Regulation of left atrial activation function to adapt PH caused by MS

The pathophysiology of this PH is shown in Fig. 14. In the development of MS, the resistance of blood flowing from the left atrium to the left ventricle gradually increases. Blood stasis in the left atrium results in an increased volume and pressure in the left atrium. The increased pressure reverses to the pulmonary veins, leading to an increase in pulmonary venous pressure, which in turn leads to an increase in pulmonary artery pressure. According to previous studies, P-V loop in left atrium changed in the progress of PH [52]. Therefore, the authors propose modified activation function for the left atrium, see in Fig. 15, and it is expressed by the sum of Gaussian functions and a linear function,

50 $$ {e}_{la\_ MS}(t)=\left(\sum \limits_{i=1}^3{X}_i(t)\times \exp\;\left[-0.5\times {\left(\frac{{\left[t\right]}_T-{Z}_i}{Y_i}\right)}^2\right]\right)+{k}_{x1}\times t, $$

where *k*_*la*_ is the linear coefficient. As the disease progresses, the magnitude of the first peak of the activation function has no change, but it becomes wide over time. The magnitude of the second peak gradually increases over time. The parameters to control the activation function could be written as

51a $$ {X}_1(t)={X}_{1,0}-{k}_{x1}\times t, $$

51b $$ {X}_2(t)={X}_{2,0}+{k}_{x2}\times t, $$

51c $$ {X}_3(t)={X}_{3,0}+{k}_{x3}\times t, $$

where*k*_*x*1_, *k*_*x*2_, and *k*_*x*3_ are the coefficients. *X*_1, 0_, *X*_2, 0_, and *X*_3, 0_ are the constants to control the magnitude of the peaks.

The P-V relations of proximal, distal pulmonary arteries, and pulmonary veins are given in Eqs. (33a, 33b, 33c, 33d, 33e, 33f), and the coefficients indicating increasing rates of the parameters *K*(*t*) are defined as *k*_*ms* _ *p*_, *k*_*ms* _ *d*_, and *k*_*ms* _ *v*_, respectively. The right ventricular compensation is given by Eq. (37), and the coefficients indicating increasing rates of the parameter *E*_*es* _ *rv*_(*t*) is defined as *k*_*rv* _ *m*_. The simulation results for the occurring and development of PH caused by MS are shown in section “Simulation Results of PH Caused by Mitral Stenosis”.

## Computer Simulation Results

In this study, the simulation time is set as 700 s, and the cardiac cycle is set as 0.7845 s (heart rate is about 76.5 beats per minute). The time step size in numerical solution is 0.0005 s. The total blood volume in the circulation system is set as 4711 ml. Sympathetic frequencies (*F*_*con*_,*F*_*Hrs*_,*F*_*vaso*_) and vagal frequency *F*_*Hrv*_ are all set as 0.5. The initial values for blood volume of each capacitor, and the current of each inductor in the platform are all given in Appendix A, as well as the values of capacitances, inductances, and resistances. The authors assume that the time-varying parameters have no change within a cardiac cycle and have increment or reduction between adjacent cycles.

### Simulation Results of PH Caused by Distal Pulmonary Artery Stenosis

**Fig. 16:**
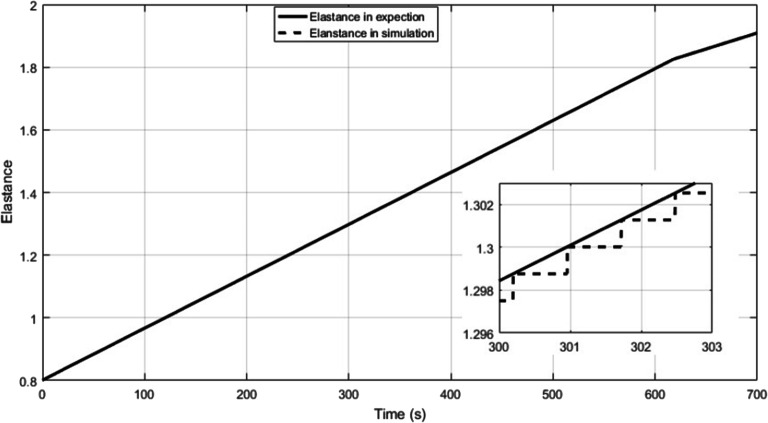
Time-varying *E*_*es* _ *rv*_ used in this simulation

The P-V relations of proximal left and right pulmonary arteries are given by Eqs. (27a)–(27b), and the values of *K*_*rpap*, 0_, *K*_*lpap*, 0_, *V*_*m*, *rpap*_, and *V*_*m*, *lpap*_ are shown in Table 8. Equations (28a, 28b) gives that *E*_*es* _ *rv*_ increases linearly over time. Clinical observations indicate that this PH case develops in a continuous way in time scale of month even year. Hence, it is reasonable to assume that the time-varying *E*_*es* _ *rv*_ keeps no change within a cardiac cycle and has an increment between cycles. The change process in *E*_*es* _ *rv*_ over simulation time is shown in Fig. 16, where the solid line is the elastance in expectation and the dash line is the elastance in simulation. The increment between cycles is small and time step size in numerical solution is tiny (0.0005 s). Hence, do not worry about the piece-wise effect. The assumption is helpful to code designer because he/she need not consider the variation within a cardiac cycle. *E*_*es* _ *rv*, 0_ = 0.8*mmHg*/*ml*. The coefficients *k*_1_, *k*_2_ and the parameters *τ*_0_, *σ* in Eq. (21b) are given in Table 8.

**Table 8:** Parameters in the simulation of DPAS

Parameter	Value	Parameter	Value
*K*_*rpap*, 0_	20 mmHg	*V*_*m*, *rpap*_	100 ml
*K*_*lpap*, 0_	20 mmHg	*V*_*m*, *lpap*_	100 ml
*k*_1_	0.0013	*k*_2_	0.0008
*r*_0_	1	*g*_*r*_	0.018
*τ*_0_	0.54 s	*σ*	0.0008

**Fig. 17:**
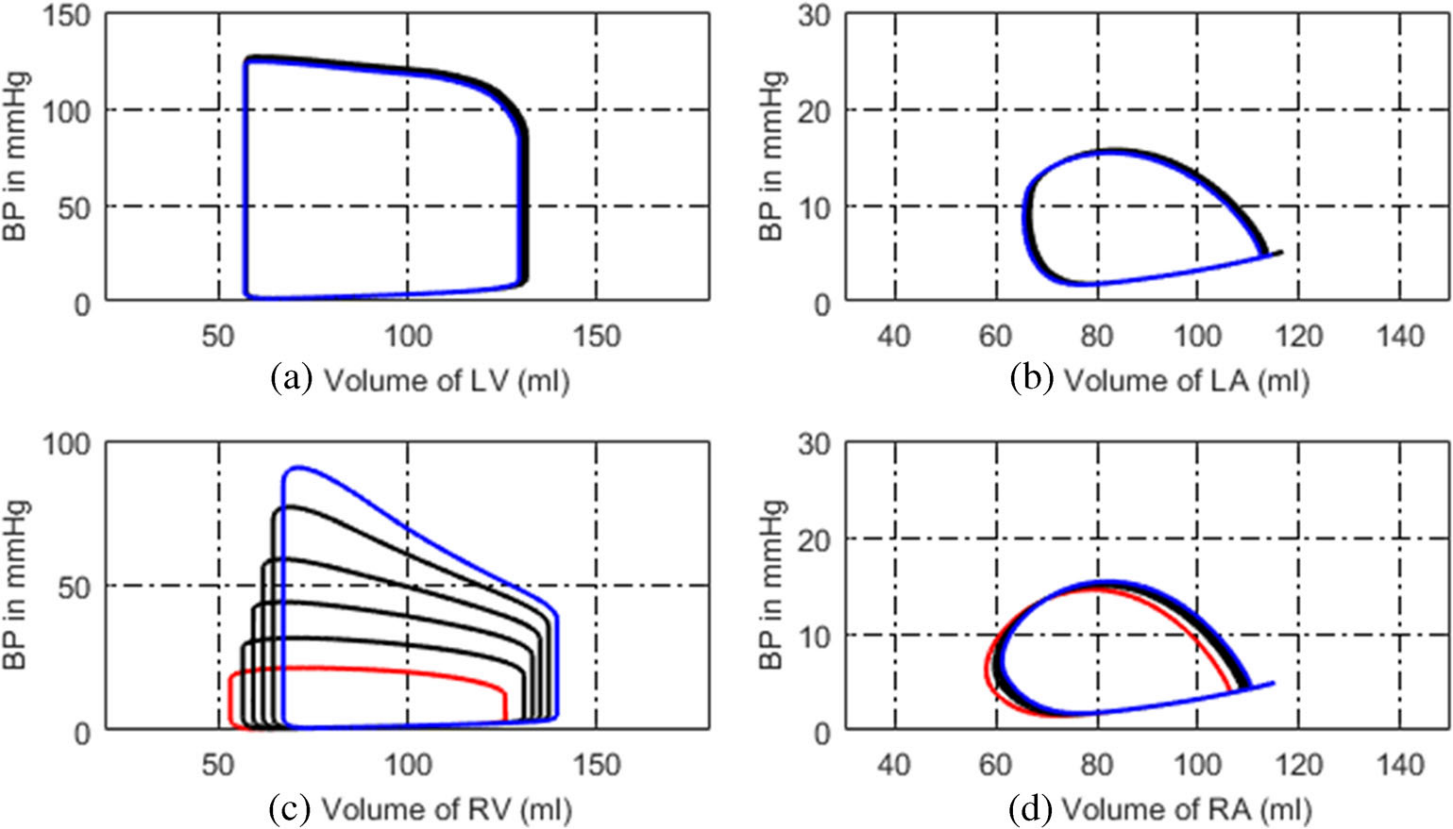
P-V loops of four chambers for PH caused by DPAS. The red loops are normal. The black ones are for developing PH and the blue ones show the late PH stage. **a** P-V loops of left ventricle. **b** P-V loops of left atrium. **c** P-V loops of right ventricle. **d** P-V loops of right atrium

**Fig. 18:**
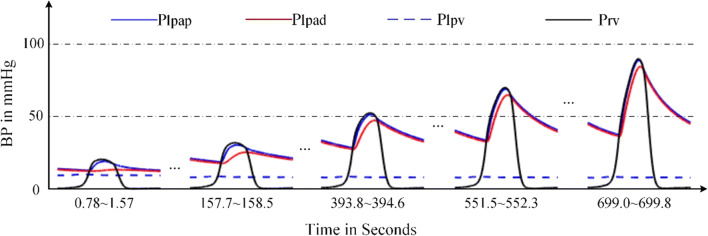
Development of the key pulmonary blood pressures for PH caused by DPAS

In the simulation of DPAS, the pathological mechanism is to increase the resistances of distal pulmonary arteries over time. The P-V relations of proximal, distal pulmonary arteries are changed, and the right ventricle compensation is added to simulate the development from health to PH. The obtained P-V relations of the four heart chambers and the output of blood pressures of vessels in the pulmonary circulation are shown in Figs. 17a–d and 18. Compared these with the normal hemodynamic conditions, it can be found that the systolic blood pressure of right ventricle continues to increase to 90 mmHg. Thus, the increased pulmonary artery pressure is high enough to push the flow of blood in the pulmonary circulation forward. As shown in Fig. 18, an increase in the resistance of the distal pulmonary artery directly leads to an increase in the blood pressure of distal pulmonary artery. The blood pressure rising in *P*_*lpap*_ can overcome the increase in *P*_*lpad*_ to make the blood in the artery to flow forward.

The model results are coincident with previous clinical observation [20, 53–55]. As can be seen from Table 2 in [53], the right ventricular pressure of five children with either stenosis or hypoplasia of both right and left pulmonary arteries raised up to 105.3 ± 37.4 (mean ± SD) mmHg in pre-dilation; however, the pressure decreased to 83.8 ± 28.6 mmHg in post-dilation. In Table 3 of [54], RV/Ao (the ratio of systolic RV pressure to aortic pressure) of the patients with branch pulmonary artery stenosis was 80.6% in pre-dilation of primary balloon angioplasty, and 85.8% in pre-dilation primary stent implantation; however, the ratio reduced to 65.9% in the second intervention. In [20, 55], it was found that the stenosis of the pulmonary artery branches and pulmonary artery led to an increase in pulmonary arterial pressure. The reference [55] gave a case of a patient of aortoarteritis with severe proximal right pulmonary artery stenosis. Hemodynamic measurement demonstrated elevated main pulmonary artery pressure of 80/24(52) mmHg. The reference [20] gave a case of pathology of pulmonary hypertension and bilateral pulmonary artery stenosis, showing pulmonary arterial pressure of 95/15 (mean 45) mmHg, and right ventricular pressure of 100/10 (mean 45) mmHg. These previous studies proved that the stenosis was the cause of PH and PH developed with the stenosis. Figures 17 and 18 illustrate the progress of PH caused by DPAS.

### Simulation Results of PH Caused by Left Ventricular Diastolic Dysfunction

The P-V relations of proximal, distal pulmonary arteries and pulmonary veins are given in Eqs. (33a, 33b, 33c, 33d, 33e, 33f). The values of *K*_*rpad*, 0_, *K*_*lpad*, 0_, *K*_*rpap*, 0_, *K*_*lpap*, 0_, *K*_*rpv*, 0_, *K*_*lpv*, 0_, *V*_*m*, *rpad*_, *V*_*m*, *lpad*_, *V*_*m*, *rpap*_,*V*_*m*, *lpap*_, *V*_*m*, *rpv*_, and *V*_*m*, *lpv*_ are shown in Table 9. The values of adjustable parameters in the case of PH caused by LVDD are given in Table 10. The modified activation function of left atrium consists of ten Gaussian functions by Eqs. (41) and (42), and initial values of parameters in the left atrial activation function are given in Table 11 and Table 12.

**Table 9:** Values of parameters *K* and *V*_*m*_ in the model of LVDD

Parameter	Value	Parameter	Value	Parameter	Value
*K*_*rpap*, 0_	20 mmHg	*K*_*lpap*, 0_	20 mmHg	*K*_*rpad*, 0_	15 mmHg
*V*_*m*, *rpap*_	100 ml	*V*_*m*, *lpap*_	100 ml	*V*_*m*, *rpad*_	150 ml
*K*_*lpad*, 0_	15 mmHg	*K*_*rpv*, 0_	5 mmHg	*K*_*lpv*, 0_	5 mmHg
*V*_*m*, *lpad*_	150 ml	*V*_*m*, *rpv*_	180 ml	*V*_*m*, *lpv*_	180 ml

**Table 10:** Values of adjustable parameters in the model of LVDD

Parameter	Value	Parameter	Value	Parameter	Value
*k*_3_	0.004	*k*_4_	0.00001	*k*_5_	0.035
*k*_6_	0.035	*k*_7_	0.02	*k*_8_	0.0012
*k*_9_	0.0004	*k*_10_	0.0008	*k*_11_	0.0000023
*τ*_*rpap* _ 0_	0.075 s	*τ*_*lpap* _ 0_	0.075 s	*τ*_*rpad* _ 0_	0.54 s
*τ*_*lpad* _ 0_	0.54 s	*τ*_*rpv* _ 0_	1.05 s	*τ*_*lpv* _ 0_	1.05 s
*g*_*cpl*_	3 ml/mmHg	*g*_*cdl*_	14 ml/mmHg	*g*_*cvl*_	20 ml/mmHg
*h*_*cpl*_	0.035 mmHg^−1^	*h*_*cdl*_	0.031 mmHg^−1^	*h*_*cvl*_	0.03 mmHg^−1^
*σ*_*l* _ *rpap*_	0.00055	*σ*_*l* _ *lpap*_	0.00055	*σ*_*l* _ *rpad*_	0.0004
*σ*_*l* _ *lpad*_	0.0004	*σ*_*l* _ *rpv*_	0.00005	*σ*_*l* _ *lpv*_	0.00005

**Table 11:** Values of parameters in the left atrial activation function

Parameter	Value	Parameter	Value	Parameter	Value
*k*_*a*1_	0.00044	*k*_*a*2_	0.000188	*k*_*a*3_	− 0.000655
*k*_*a*4_	0.0001	*k*_*a*5_	0.0003	*k*_*a*6_	0.000278
*k*_*a*7_	0.000431	*k*_*a*8_	0.000275	*k*_*a*9_	0.000502
*k*_*a*10_	0.000055

**Table 12:** Initial values of parameters in the left atrial activation function

Parameter	*i* = 1	*i* = 2	*i* = 3	*i* = 4	*i* = 5	*i* = 6	*i* = 7	*i* = 8	*i* = 9	*i* = 10
*a*_*i*, 0_	0	0	0.9	0	0	0	0	0	0	0
*b*_*i*_	0.12	0.09	0.038	0.07	0.09	0.05	0.04	0.08	0.1	0.04
*c*_*i*_	0.005	0.08	0.14	0.25	0.31	0.375	0.45	0.62	0.784	0.7845

**Fig. 19:**
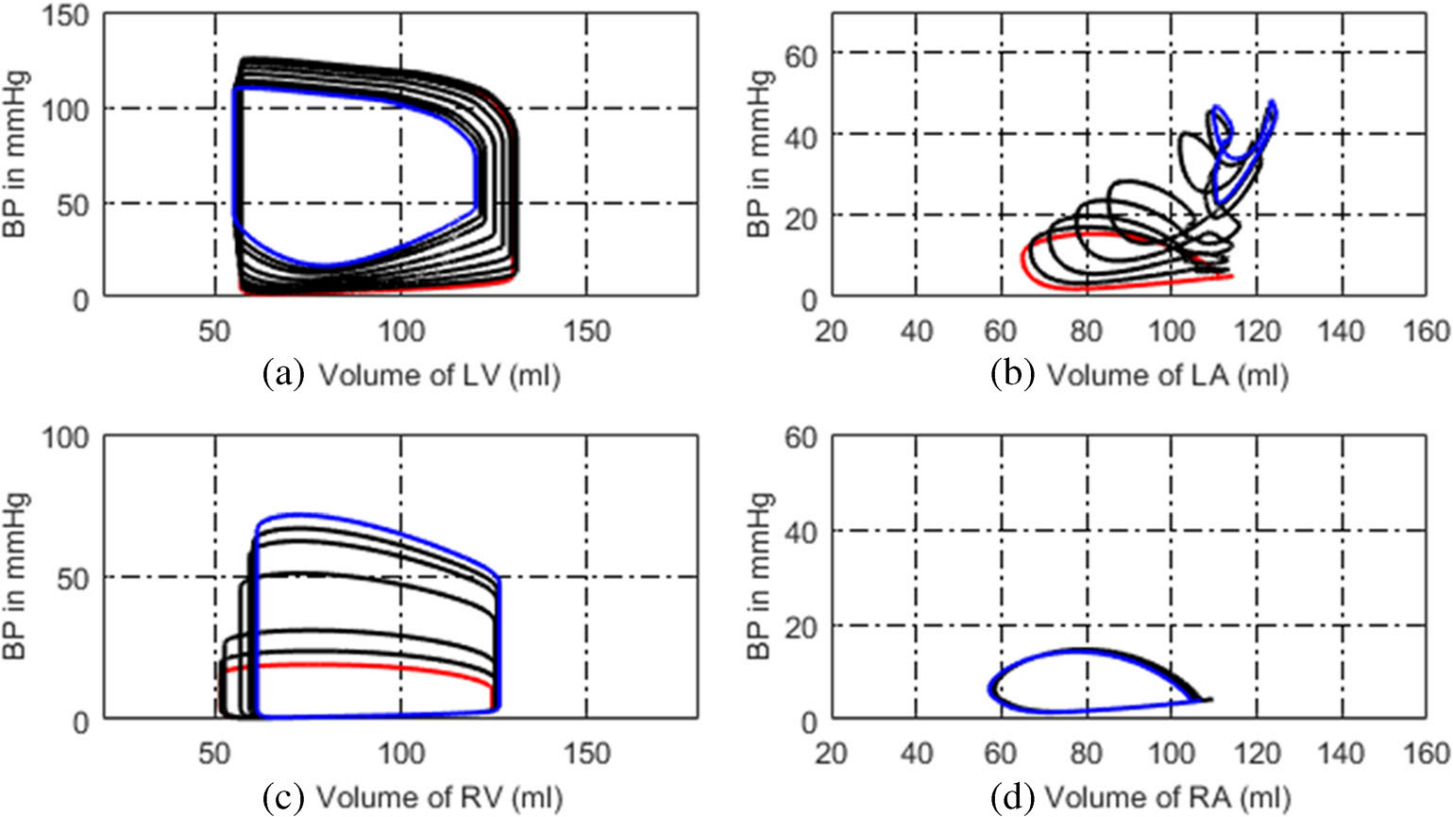
P-V loops of four chambers for PH caused by LVDD. The red loops are for normal, the black ones are for developing PH and the blue ones are for this PH at late stage. **a** P-V loops of left ventricle. **b** P-V loops of left atrium. **c** P-V loops of right ventricle. **d** P-V loops of right atrium

**Fig. 20:**
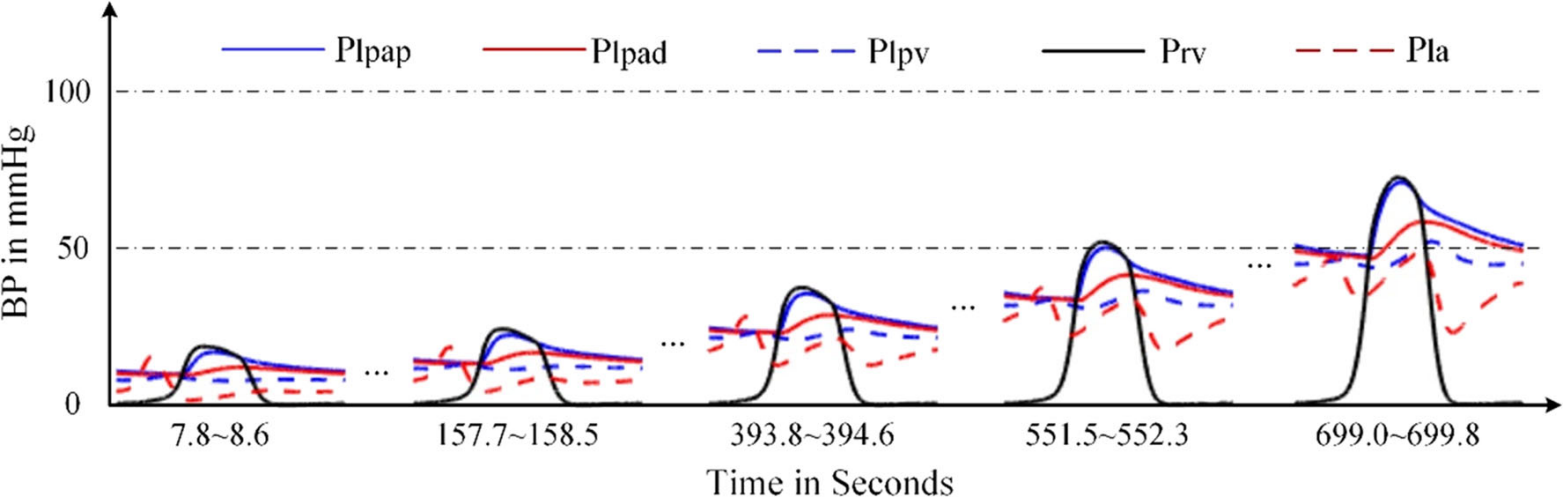
Development of key pulmonary blood pressures for PH due to LVDD

In the model of LVDD, the left ventricular end-diastolic pressure is increased by linearly increasing the parameters of *M*_*lv*_ and *λ*_*lv*_. The P-V relations of vessels in the pulmonary circulation are changed, and the PVR is increased too. The P-V relation and activation function of the left atrium are revised to compensate for the increased left atrial pressure and volume. The simulation results are shown in Figs. 19a–d and 20. Compared these with the normal hemodynamic conditions, the left ventricular diastolic dysfunction leads to an increased left ventricular end-diastolic pressure. The left atrium needs to increase the pressure to ensure the blood returns to the left ventricle. Long-term blood return is blocked, causing blood to accumulate in the left atrium and its volume increase consequently. The right ventricular systolic pressure would increase to overcome the increased pulmonary artery pressure. As shown in Fig. 20, the blood pressures in pulmonary vessels are also increased. Compared these with the simulation results of DPAS model, the pulmonary vein pressure is higher than that in PH caused by DPAS. The reason is in the mechanism of PH. For a LVDD case, the pressure in the left atrium and the pulmonary veins rises, which in turn leads to an increase in the distal pulmonary artery blood pressure.

Figure 19 illustrates the P-V loops of the left ventricle, left atrium, right ventricle, and right atrium in the model of LVDD. The direct manifestation of left ventricular diastolic dysfunction is to increase the left ventricular end-diastolic pressure, which is shown in Fig. 19a. These simulation results of this paper could be validated by previous studies. In the reference [28, 36], the schematic diagram of P-V relations in systolic heart failure and in pathologies with diastolic dysfunction were collected from patients. The P-V loop shifted to upward and left, which is shown in Fig. 5 of [28] and Fig. 2 of [36]. As is seen in Fig. 19b, the volume of left atrium increases. In [30], the data showed similar observation where increased LA volume for patients with diastolic heart failure in comparison with to normal control group was illustrated. With this PH development, the P-V loop of the left atrium becomes two loops, and the systolic blood pressure of left atrium continues to rise. This is because long-term obstruction of blood flow changes the function and structure of the left atrium. The clinical data in [33] showed similar results, where the pressure and volume of the left atrium in HFpEF (heart failure with preserved ejection fraction) increased, and left atrial stiffness also increased compared with the control group, which are shown in Fig. 1 and Table 2 of [33]. Figure 20 displays the changes in pulmonary artery and pulmonary venous pressure. The increase in left ventricular end-diastolic pressure affects the pressure in the left atrium and the pulmonary veins, which in turn affects the pressure in the pulmonary arteries and ultimately leads to an increase in the right ventricular systolic blood pressure.

### Simulation Results of PH Caused by Ventricular Septal Defect

In the simulation of PH caused by VSD, the P-V relations of proximal, distal pulmonary arteries and pulmonary veins are given in Eqs. (33a, 33b, 33c, 33d, 33e, 33f). The values of *K*_*rpad*, 0_, *K*_*lpad*, 0_, *K*_*rpap*, 0_, *K*_*lpap*, 0_, *K*_*rpv*, 0_, *K*_*lpv*, 0_, *V*_*m*, *rpad*_, *V*_*m*, *lpad*_, *V*_*m*, *rpap*_, *V*_*m*, *lpap*_, *V*_*m*, *rpv*_, and *V*_*m*, *lpv*_ are shown in Table 9. The values of adjustable parameters in the case of PH caused by VSD are given in Table 13. The modified activation function of left atrium consists of three Gaussian functions by Eqs. (44) and (45a, 45b, 45c), and its initial values of parameters in the activation function are given in Table 14.

**Table 13:** Values of adjustable parameters in the model of VSD

Parameter	Value	Parameter	Value	Parameter	Value
*k*_*α*2_	0.000056	*k*_*α*3_	0.000056	*k*_*r*_	0.0408
*k*_*vsd* _ *p*_	0.035	*k*_*vsd* _ *d*_	0.035	*k*_*vsd* _ *v*_	0.035
*R*_*ltor*, 0_	100 mmHg s ml^−1^	*g*_*cpv*_	3 ml/mmHg	*g*_*cdv*_	14 ml/mmHg
*g*_*cvv*_	20 ml/mmHg	*h*_*cpv*_	0.035 mmHg^−1^	*h*_*cdv*_	0.035 mmHg^−1^
*h*_*cvv*_	0.03 mmHg^−1^	*σ*_*v* _ *rpap*_	0.00015	*σ*_*v* _ *lpap*_	0.00015
*σ*_*v* _ *rpad*_	0.00008	*σ*_*v* _ *lpad*_	0.00008	*σ*_*v* _ *rpv*_	0.00002
*σ*_*v* _ *lpv*_	0.00002	*k*_*rv* _ *v*_	0.0012	*k*_*la* _ *v*_	0.0003

**Table 14:** Initial value of parameters in the activation function of left atrium

Parameters	*i* = 1	*i* = 2	*i* = 3
*α*_*i*, 0_	0.9	0	0
*β*_*i*_	0.038	0.05	0.05
*ω*_*i*_	0.145	0.37	0.42

**Fig. 21:**
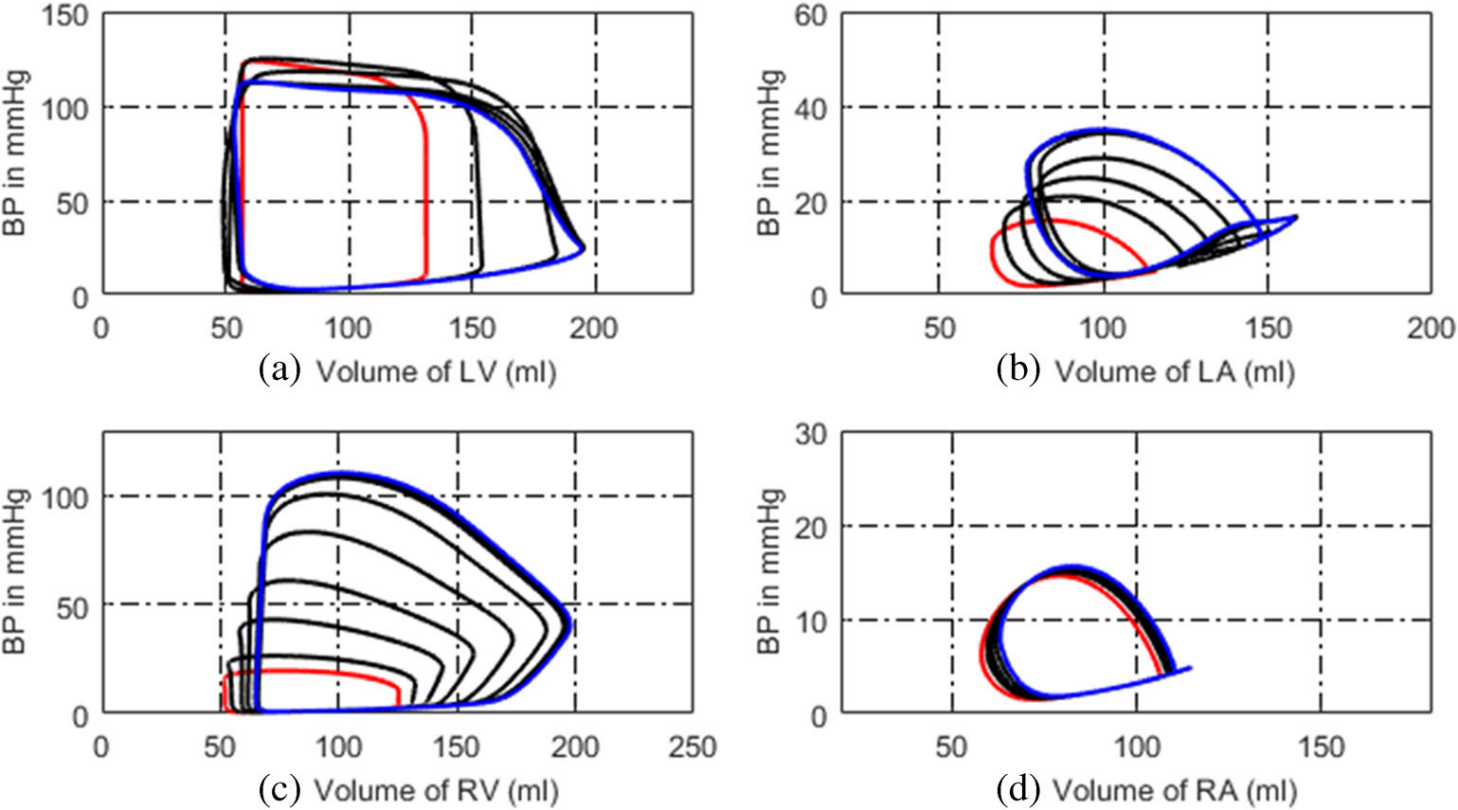
P-V loops of the four chambers for PH development caused by VSD. The red loops are for normal, the black ones are for developing PH and the blue ones are for the late PH at serious condition. **a** P-V loops for left ventricle. **b** P-V loops for left atrium. **c** P-V loops for right ventricle. **d** P-V loops for right atrium

**Fig. 22:**
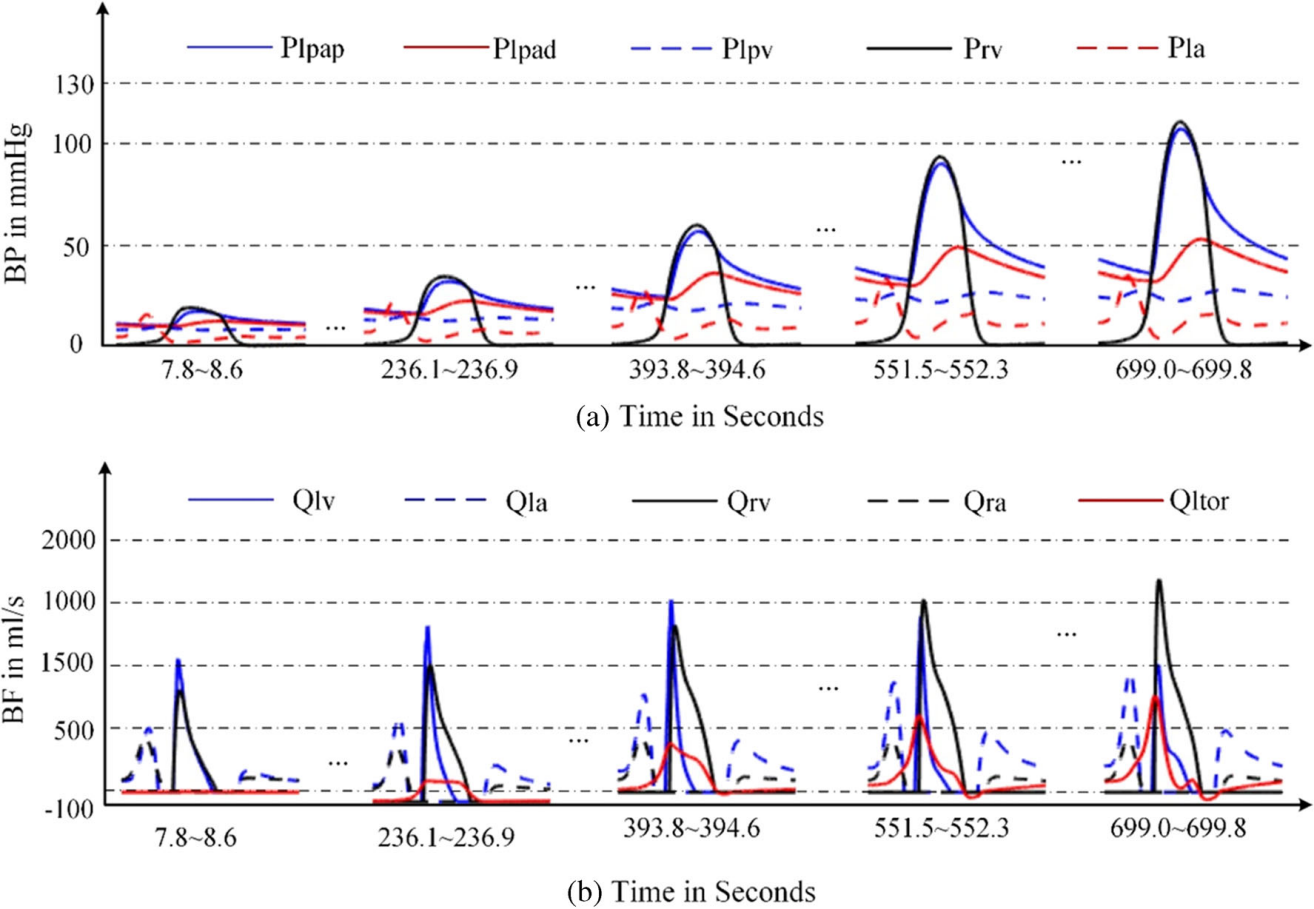
Blood pressures and blood flows in PH development caused by VSD. **a** Key blood pressures in the pulmonary circulation. **b** Corresponding blood flows

A branch consisting of a resistor *R*_*ltor*_ is used to model blood flow caused by VSD. The decreasing *R*_*ltor*_ over cardiac beat number simulates the development of VSD. Both right ventricle and left atrium increase contractibility to adapt the abnormal hemodynamics. Comparing these with those in normal hemodynamic conditions, both the volume and SV of the left ventricle increase greatly; meanwhile, the systolic pressure decreases. However, the SV of right ventricle increases. Thus, the SVs of left and right ventricles are out of balance because of the VSD branch flow. The left atrium regulates its pumping function to maintain the circulation system working. Figure 21 gives the hemodynamics of the four heart chambers of PH caused by VSD. The corresponding blood pressures of various pulmonary vessels in the progress of PH are shown in Fig. 22a. As seen in Fig. 22b, the blood flow between the left and right ventricle, *Q*_*ltor*_, increases over time. Positive value of *Q*_*ltor*_ means flow from left to right, and negative value means the contrary. It is seen from Fig. 22b that the blood flow is unidirectional from left to right at the beginning of VSD. As time goes on, the VSD becomes serious as seen at simulation time greater than 500 s. There is small negative blood flow from right ventricle to left in very short time interval. This PH caused by VSD develops finally into obstructive PH. The flow could be bidirectional and/or right-to-left. However, the further development of this PH is not considered in this study.

The changes of P-V loops for four heart chambers are given in Fig. 21. Abnormal flow between the ventricles results in volume overload of the left atrium and both ventricles, which were also reported in the reference [43]. Figure 21 a shows a decrease in systolic blood pressure in the left ventricle; Fig. 21 c shows an increase in systolic blood pressure in the right ventricle, as well as the pressure in pulmonary artery, which are shown in Fig. 22a. The results we simulate for the VSD are close to what are reported in Table 2, and Figs. 3, 4, and 5 of [11] where the authors conducted the simulation of Eisenmenger syndrome with VSD. The results in [11] showed that there was a remarkable increase in the pressures at pulmonary artery and right ventricle; however, the left ventricular pressure and pulmonary compliance decreased.

### Simulation Results of PH Caused by Mitral Stenosis

In this simulation, the P-V relation of proximal, distal pulmonary arteries, and pulmonary veins are given in Eqs. (33a, 33b, 33c, 33d, 33e, 33f). The values of *K*_*rpad*, 0_, *K*_*lpad*, 0_, *K*_*rpap*, 0_, *K*_*lpap*, 0_, *K*_*rpv*, 0_, *K*_*lpv*, 0_, *V*_*m*, *rpad*_, *V*_*m*, *lpad*_, *V*_*m*, *rpap*_, *V*_*m*, *lpap*_, *V*_*m*, *rpv*_, and *V*_*m*, *lpv*_ are shown in Table 9. The values of adjustable parameters in the model of MS are given in Table 15. The modified activation function of left atrium consists of three Gaussian functions by Eqs. (50) and (51a, 51b, 51c), and initial values of parameters in the left atrial activation function are given in Table 15 and Table 16.

**Table 15:** Values of the parameters in the model of MS

Parameter	Value	Parameter	Value	Parameter	Value
*k*_12_	0.0003	*k*_13_	0.0003	*k*_14_	0.001
*k*_15_	0.0000056	*k*_*x*1_	0.000336	*k*_*x*2_	0.000168
*k*_*x*3_	0.000168	*k*_*ms* _ *p*_	0.035	*k*_*ms* _ *d*_	0.035
*k*_*ms* _ *v*_	0.035	*g*_*cpm*_	3 ml/mmHg	*g*_*cdm*_	14 ml/mmHg
*g*_*cvm*_	20 ml/mmHg	*h*_*cpm*_	0.035 mmHg^−1^		0.035 mmHg^−1^
*h*_*cvm*_	0.03 mmHg^−1^	*σ*_*m* _ *rpap*_	0.00075	*σ*_*m* _ *lpap*_	0.00075
*σ*_*m* _ *rpad*_	0.0006	*σ*_*m* _ *lpad*_	0.0006	*σ*_*m* _ *rpv*_	0.0005
*σ*_*m* _ *lpv*_	0.0005	*k*_*rv* _ *m*_	0.0012

**Table 16:** Initial value of parameters in the activation function of left atrium

Parameter	*i* = 1	*i* = 2	*i* = 3
*X*_*i*, 0_	0.9	0	0
*Y*_*i*_	0.038	0.05	0.05
*Z*_*i*_	0.145	0.37	0.42

**Fig. 23:**
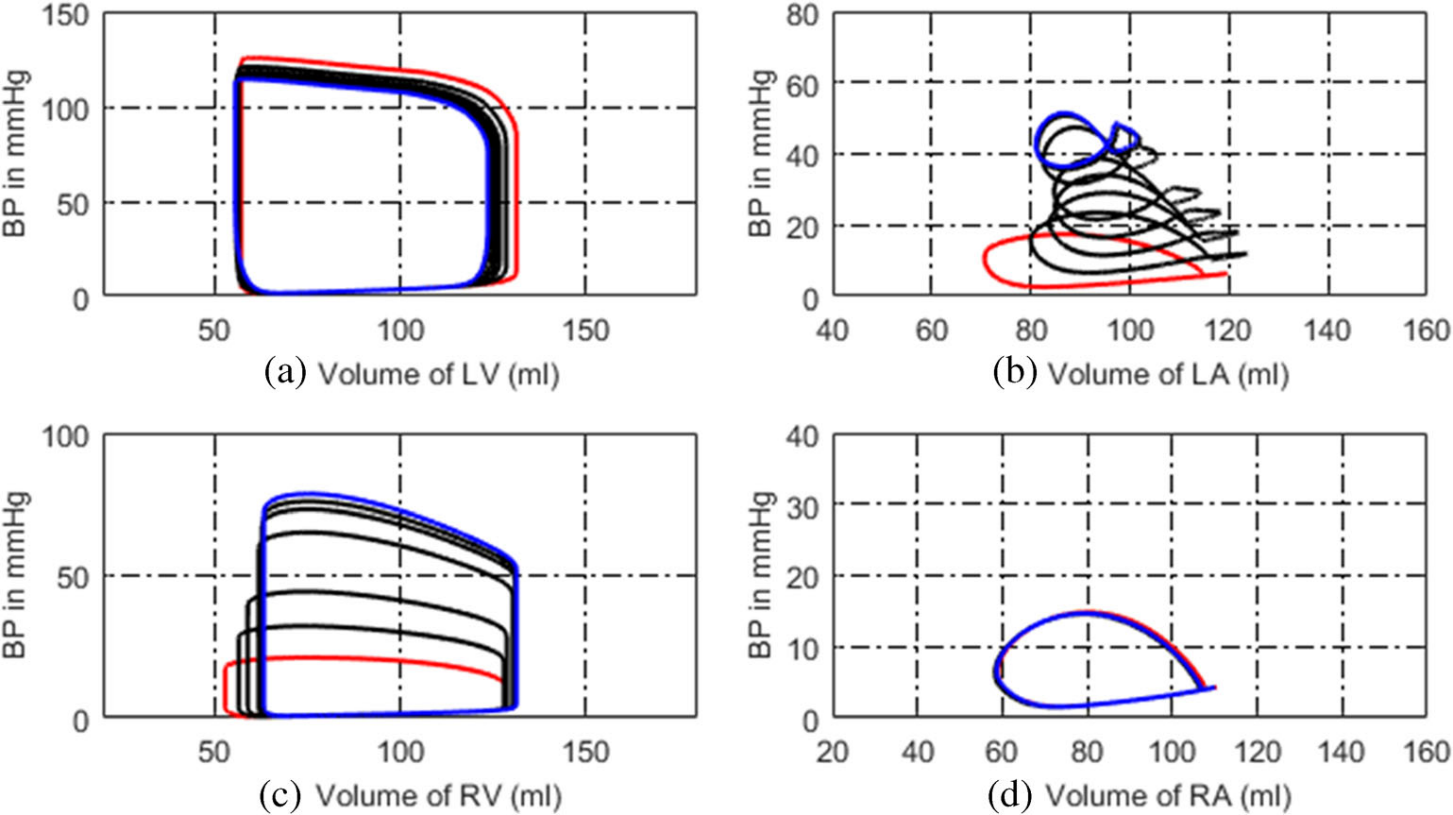
P-V loop of four chambers during PH development caused by MS. The red loops are for the beginning of PH, the black ones are for developing PH and the blue ones present the late stage of this PH. **a** P-V loops for left ventricle. **b** P-V loops for left atrium. **c** P-V loops for right ventricle. **d** P-V loops for right atrium

**Fig. 24:**
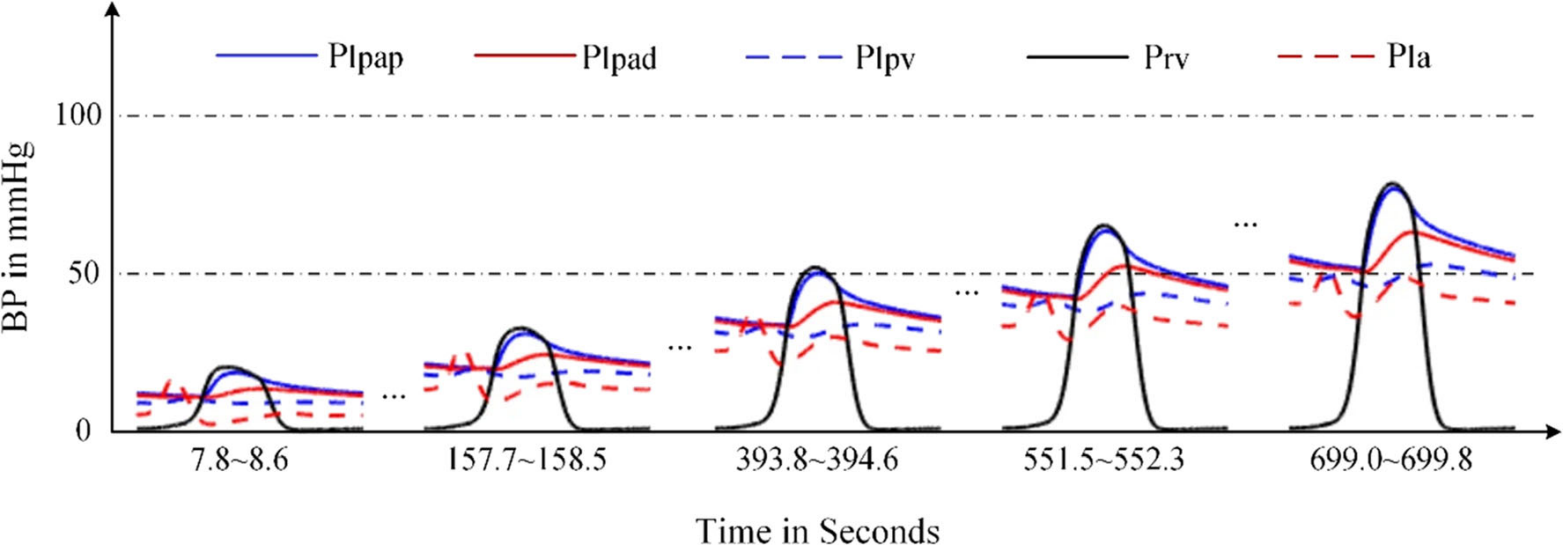
Development of key pulmonary blood pressures for PH due to MS

The increasing resistance *R*_*m*_ is used to simulate the pathological mechanism of MS. The P-V relations of vessels in the pulmonary circulation, increasing of PVR, contractibility of the right ventricle, and left atrium are all together adapted to the abnormal hemodynamic flow caused by MS. The simulation results are shown in Fig. 23a–d. Compared these with the normal hemodynamics, the P-V loop of left ventricular remains almost no change, and stroke volume decreases slightly. The left atrial systolic and diastolic pressures increase, and the P-V loop of left atrium has two loops. The pressures of key pulmonary vessels in this PH development are shown in Fig. 24.

The changes of P-V loops for the heart chambers are given in Fig. 23. Pathological manifestations of mitral stenosis show that the flow from the left atrium to left ventricle is impeded. In the platform in Fig. 1, mitral stenosis is simulated by increasing resistance *R*_*m*_. The clinical data collected from patients showed that the mitral resistance was significantly exponential and inverse to mitral area [48], see Fig. 1 of this reference. It can be seen from Fig. 23b that the P-V loop of the left atrium has changed, and both the volume and pressure of left atrium increase. This phenomenon was showed in [52] where, compared with the control group, the pressure and volume of the left atrium of the mitral stenosis group was significantly increased. See Fig. 5 of [52]. The changes of P-V loops for the left and right ventricles are given in Fig. 23 a and c. The systolic blood pressure of the left ventricle decreases slightly; however, the systolic blood pressure of the right ventricle increases to 78 mmHg, and these results agree with those in [50, 51]. Figure 24 shows the changes of pulmonary artery pressures in the model of mitral stenosis, and an increase in pulmonary artery pressure is a manifestation of pulmonary hypertension.

## Discussions

### Explanation of the Four Typical Case in Pathogenesis

PVR is an important indicator for pulmonary hemodynamics. The irreversible injury, the intima, and medial thickening of the vessels lead to thickening of the blood vessel wall and narrowing of the lumen. PVR increases, which may results in PH ultimately. It can be seen from the simulation of DPAS that the pulmonary vascular stenosis leads to increasing resistance at the distal pulmonary arteries and hence results in PH. In the PHs caused by LVDD, VSD, and MS, PVR may be normal in the early stage of PH, but become obviously increasing with elevating pulmonary artery pressure.

From a physiological point of view, the morphology and structure of right and left ventricle are to adapt their functional requirements for pumping blood. The right ventricle could be considered as a sidewall that attaches additional muscles to the left ventricular wall. The wall of the right ventricle is much thinner than that of the left ventricle. Therefore, it cannot maintain normal contractile function when the mPAP increases. However, it can be well adapted to the increase of blood volume due to right ventricular reflux. When the right ventricular afterload increases rapidly, it can cause a significant expansion of the right ventricle. However, if the mPAP increases gradually over a long time, the right ventricle reforms to ventricular hypertrophy by increasing the thickness of one side of the wall to meet the needed contractile force. As a result, the right ventricle can accommodate a sustained and significant increase in mPAP. A question is that the mPAP rises usually faster than the right ventricle adaptability, so the contractility does not meet the needed force, which leads obstacles in the right ventricular motor function. In the simulation of the four typical cases of PH, it is found that the P-V loop of right ventricle gradually changes from the normal to a P-V loop with very high systolic pressure, and the right ventricle volume increases with the disease development.

**Fig. 25:**
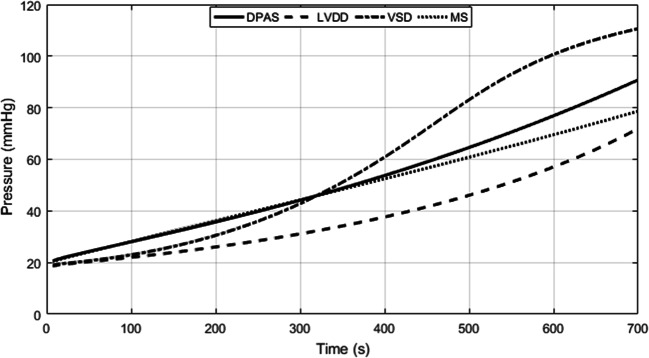
Development of systolic RV pressure of the typical PH cases with respect to time

The systolic pressures of the typical PH cases at right ventricle develop with time in our simulated conditions, see Fig. 25. The abnormal hemodynamics of PH successfully occurs and develops with the typical causes. The increasing rates of the pressure with respect to time are obviously nonlinear due to the complex interplay among heart, systemic and pulmonary vessels even if the causes are linearly varying with time. Therefore, the nonlinearity of pressure varying would be heavier if the causes vary nonlinearly.

### Clinical Significance of This Study

The platform and simulation results could have potential applications and/or clinical significances.

- The platform could be helpful for educating senior students and new physicians to understand how a PH case develops with a typical cause.
- The platform could be applicable for evaluating how fast a PH case develops if the cause changes nonlinear with respect to time. Some linear equations with time are used in the simulations because of the absence of accurate knowledge. If necessary, it is convenient for an operator to watch how the circulation system responds to a speeded-up cause. Based on the platform, it is also possible to study how the circulation system responds if multiple causes occur simultaneously.
- These simulations could be helpful for physicians to understand how heart chambers, systemic and pulmonary vessels regulate their functions to adapt to abnormal hemodynamics in different PH case development. For example, in a PH case caused by DPAS, the right ventricle and pulmonary vessels tunes P-V relation to adapt the increasing resistance induced by artery stenosis; however, the other three chambers and systemic vessels have little change. As a comparison, in a PH case caused by LVDD, both the left atrium and right ventricle tune the P-V relations, but the right atrium has little change.
- The simulation results could be helpful for a physician in directive guidance for further examination, even helpful in identification of a cause. A physician can obtain some hemodynamic knowledge via auscultation, echocardiography, chest radiography and high-resolution CT. With these simulation results in mind, the patient could be guided to specific further examination.

## Conclusions

A lumped-parameter platform consisting of analog circuit elements for simulating human circulation system is set up in this study. The developments of four typical cases of PH caused by different pathogenesis are separately simulated in the platform. On PH caused by distal pulmonary artery stenosis, the thick and stiff distal arteries are modeled by increasing resistances. On PH due to left ventricular diastolic dysfunction, PH develops under the proposed model, showing the decrease of left ventricular myocardial compliance and filling disorder. On PH caused by ventricular septal defect, a branch is proposed to simulate the branch flow between the left and right ventricles. On PH caused by mitral stenosis, an increasing resistor is used to simulate the degree of stenosis. For each PH development, the regulation rules for heart chambers, arteries, and veins are proposed to adapt to the hemodynamic abnormalities. The simulation results are close to clinical investigations. These works could be powerful to understand the causes that lead to PH and regulation mechanism in PH development.

### B. Electronic supplementary material

ESM 1 (DOCX 14 kb)

## A. Appendix. Parameters and Initial Conditions of the Normal Human Circulation System Circuit

The values of parameters for the normal human circulation system circuit model are given in Tables 17, 18, and 19**.** The initial conditions of the blood volumes in four chambers, vessels, and the blood flows in the inductors are shown in Table 20. These values are from previous studies [14, 15] and slightly tuned when necessary.

**Table 17:** Resistors and diodes of the normal human circulation system circuit model [14, 15]

Parameter	Description	Value
*Flow resistances*		
*R*_*m*_	Mitral valve	0.02 mmHg s ml^−1^
*R*_*a*_	Aortic valve	0.02 mmHg s ml^−1^
*R*_*haa*_	Head and arm artery	8 mmHg s ml^−1^
*R*_ln*a*_	Left neck artery	12 mmHg s ml^−1^
*R*_lc*a*_	Left clavicular artery	12 mmHg s ml^−1^
*R*_*aop*_	Proximal aorta	1.2 mmHg s ml^−1^
*R*_*rula*_ and *R*_*lula*_	Right (Left) upper limb artery	0.5 mmHg s ml^−1^
*R*_*rica*_ and *R*_*lica*_	Right (Left) internal carotid artery	0.5 mmHg s ml^−1^
*R*_*sap*_	Proximal systemic artery	See Eq. 11
*R*_*rsv*_	Right subclavian vein	0.27 mmHg s ml^−1^
*R*_*rijv*_ and *R*_*lijv*_	Right (Left) internal jugular vein	0.25 mmHg s ml^−1^
*R*_*lsv*_	Left subclavian vein	0.25 mmHg s ml^−1^
*R*_*sv*_	Systemic vein	0.2 mmHg s ml^−1^
*R*_*vc*_	Vena cava	See Eq. 9
*R*_*t*_	Tricuspid valve	0.03 mmHg s ml^−1^
*R*_*p*_	Pulmonary valve	0.01 mmHg s ml^−1^
*R*_*rpap*_ and *R*_*lpap*_	Right (Left) proximal pulmonary artery	0.05 mmHg s ml^−1^
*R*_*rpad*_ and *R*_*lpad*_	Right (Left) distal pulmonary artery	0.06 mmHg s ml^−1^
*R*_*rpv*_ and *R*_*lpv*_	Right (Left) pulmonary vein	0.07 mmHg s ml^−1^
*Viscoelastic resistances*		
*R*_*chaa*_	Head and arm artery	0.01 mmHg s ml^−1^
*R*_*clna*_	Left neck artery	0.01 mmHg s ml^−1^
*R*_*clca*_	Left clavicular artery	0.01 mmHg s ml^−1^
*R*_*caop*_	Proximal aorta	0.01 mmHg s ml^−1^
*R*_*crpap*_ and *R*_*clpap*_	Right (Left) proximal pulmonary artery	0.005 mmHg s ml^−1^
*R*_*crpad*_ and *R*_*clpad*_	Right (Left) distal pulmonary artery	0.005 mmHg s ml^−1^
*R*_*crpv*_ and *R*_*clpv*_	Right (Left) pulmonary veins	0.005 mmHg s ml^−1^

**Table 18:** Inductors of the normal human circulation system circuit model [14, 15]

Parameter	Description	Value
*L*_*aop*_	Proximal aorta	0.001 mmHg s^2^ ml^−1^
*L*_*rpap*_ and *L*_*lpap*_	Right (Left) proximal pulmonary artery	0.001 mmHg s^2^ ml^−1^

**Table 19:** Capacitors of the normal human circulation system circuit model [14, 15]

Parameter	Description	Value
*C*_*haa*_	Head and arm artery	0.7 ml mmHg^−1^
*C*_*lna*_	Left neck artery	0.7 ml mmHg^−1^
*C*_*lca*_	Left clavicular artery	0.7 ml mmHg^−1^
*C*_*aop*_	Proximal aorta	0.8 ml mmHg^−1^
*C*_*rula*_	Right upper limb artery	3 ml mmHg^−1^
*C*_*lula*_	Left upper limb artery	2 ml mmHg^−1^
*C*_*rica*_	Right internal carotid artery	2 ml mmHg^−1^
*C*_*lica*_	Left internal carotid artery	3 ml mmHg^−1^
*C*_*sap*_	Proximal systemic artery	See Eq. 10
*C*_*rsv*_	Right subclavian vein	9 ml mmHg^−1^
*C*_*rijv*_ and *C*_*lijv*_	Right (Left) internal jugular vein	9 ml mmHg^−1^
*C*_*lsv*_	Left subclavian vein	9 ml mmHg^−1^
*C*_*sv*_	Systemic vein	See Eq. 7
*C*_*vc*_	Vena cava	See Eq. 8
*C*_*rpap*_ and *C*_*lpap*_	Right (Left) proximal pulmonary artery	1.5 ml mmHg^−1^
*C*_*rpad*_ and *C*_*lpad*_	Right (Left) distal pulmonary artery	9 ml mmHg^−1^
*C*_*rpv*_ and *C*_*lpv*_	Right (Left) pulmonary vein	15 ml mmHg^−1^

**Table 20:** Initial conditions of the normal human circulation system circuit model [14, 15]

Compartment	Value	Compartment	Value
Total blood volume	4711 ml		
*Volume*			
Left ventricle	123 ml	Right ventricle	110 ml
Left atrium	63 ml	Right atrium	53 ml
Head and arm artery	111 ml	Left neck artery	117 ml
Left clavicular artery	117 ml	Proximal aorta	64 ml
Right upper limb artery	29 ml	Left upper limb artery	19 ml
Right internal carotid artery	20 ml	Left internal carotid artery	37 ml
Proximal systemic artery	217 ml	Systemic vein	2526 ml
Right subclavian vein	66 ml	Left subclavian vein	66 ml
Right internal jugular vein	69 ml	Left internal jugular vein	66 ml
Vena cava	170 ml	Right proximal pulmonary artery	64 ml
Left proximal pulmonary artery	64 ml	Right distal pulmonary artery	140 ml
Left distal pulmonary artery	140 ml	Right pulmonary vein	130 ml
Left pulmonary vein	130 ml		
*Flow*			
Proximal aorta	40 ml s^−1^	Right proximal pulmonary artery	16 ml s^−1^
Left proximal pulmonary artery	16 ml·s^−1^		
