# Supplementary material for: Lumped-Parameter Circuit Platform for Simulating Typical Cases of Pulmonary Hypertensions from Point of Hemodynamics
Source: J Cardiovasc Transl Res. 2020 Jan 13;13(5):826–52. doi: 10.1007/s12265-020-09953-y (PMC7541384; doi:10.1007/s12265-020-09953-y)
Supplement: Supplementary file 1 — (DOCX 14 kb) [file 12265_2020_9953_MOESM1_ESM.docx]

Supplementary Materials

The matlab codes for these simulations can be accessed at :

https://github.com/tanghongdlut/An-Analog-Circuit-Platform-for-Simulating-Typical-Pulmonary-Hypertensions-from-Point-of-Hemodynamics. Those who are interested reproducing the results can download the codes freely.
